# Mammalian lectin arrays for screening host–microbe interactions

**DOI:** 10.1074/jbc.RA120.012783

**Published:** 2020-02-24

**Authors:** Sabine A. F. Jégouzo, Conor Nelson, Thomas Hardwick, S. T. Angel Wong, Noel Kuan Kiat Lau, Gaik Kin Emily Neoh, Rocío Castellanos-Rueda, Zhiyao Huang, Benjamin Mignot, Aanya Hirdaramani, Annie Howitt, Kathryn Frewin, Zheng Shen, Rhys J. Fox, Rachel Wong, Momoko Ando, Lauren Emony, Henderson Zhu, Angela Holder, Dirk Werling, Nitya Krishnan, Brian D. Robertson, Abigail Clements, Maureen E. Taylor, Kurt Drickamer

**Affiliations:** ‡ Department of Life Sciences, Imperial College London, London SW7 2AZ, United Kingdom; § Department of Pathobiology and Population Sciences, Royal Veterinary College, North Mymms, Hatfield, Hertfordshire AL9 7TA, United Kingdom; ¶ Department of Infectious Disease and MRC Centre for Molecular Bacteriology and Infection, Imperial College London, London SW7 2AZ, United Kingdom; ‖ Department of Life Sciences and MRC Centre for Molecular Bacteriology and Infection, Imperial College London, London SW7 2AZ, United Kingdom

**Keywords:** glycobiology, lectin, carbohydrate-binding protein, carbohydrate function, lipopolysaccharide (LPS), array screening, glycan-binding receptors, innate immune system, ligand binding, pathogen

## Abstract

Many members of the C-type lectin family of glycan-binding receptors have been ascribed roles in the recognition of microorganisms and serve as key receptors in the innate immune response to pathogens. Other mammalian receptors have become targets through which pathogens enter target cells. These receptor roles have often been documented with binding studies involving individual pairs of receptors and microorganisms. To provide a systematic overview of interactions between microbes and the large complement of C-type lectins, here we developed a lectin array and suitable protocols for labeling of microbes that could be used to probe this array. The array contains C-type lectins from cow, chosen as a model organism of agricultural interest for which the relevant pathogen–receptor interactions have not been previously investigated in detail. Screening with yeast cells and various strains of both Gram-positive and -negative bacteria revealed distinct binding patterns, which in some cases could be explained by binding to lipopolysaccharides or capsular polysaccharides, but in other cases they suggested the presence of novel glycan targets on many of the microorganisms. These results are consistent with interactions previously ascribed to the receptors, but they also highlight binding to additional sugar targets that have not previously been recognized. Our findings indicate that mammalian lectin arrays represent unique discovery tools for identifying both novel ligands and new receptor functions.

## Introduction

Soluble and membrane-bound mammalian glycan-binding proteins in the C-type lectin family are responsible for recognition of microorganisms and for cell–cell interactions (1, 2). Sugar-binding activity of these receptors resides in modular carbohydrate-recognition domains (CRDs)^2^ and is based on ligation of adjacent hydroxyl groups, usually at the 3- and 4-position in a monosaccharide target, to a conserved Ca^2+^ in the primary binding site. The CRDs in the roughly 25 members of the family fall broadly into two groups: those that interact with sugars related to mannose and GlcNAc, through their equatorial 3- and 4-OH groups, and those that interact with galactose and GalNAc, in which the 4-OH group is axial. Many CRDs in the mannose-recognition group also bind to fucose. Binding to ligands depends on the presence of free OH groups. Thus, target monosaccharides are often found in the nonreducing ends of oligosaccharides. Selective binding to specific oligosaccharide ligands results from additional interactions with further sugar residues in secondary binding sites.

The C-type lectins play multiple roles in the innate immune system. The soluble C-type lectins are collectins, which contain collagen-like domains in addition to the C-type CRDs (3). Collectins are mostly found in blood and in the surfactant layer on the surface of the alveolar fluid in the lungs. These proteins aid in neutralization of pathogens by activating complement, by opsonizing proteins for uptake by complement-binding receptors on macrophages, and by causing agglutination. Membrane-bound C-type lectins are found on various cells of the innate system, including macrophages and dendritic cells (1). Many of these receptors mediate endocytosis of ligands, which can be a route for neutralizing pathogens as well as subsequently stimulating the adaptive immune response. C-type lectins involved in endocytosis are also found on hepatocytes and vascular endothelial cells (2).

Extensive genomic and biochemical analysis has provided a nearly complete account of receptors containing C-type CRDs in the human immune system, but C-type lectins from other mammals, apart from mouse, have not been as well-studied. Because there are many differences between the mouse and human immune systems, there is need to study other species. Cows are not only important agriculturally, but can also be a useful model for infectious diseases in humans (4, 5). For example, mouse models of tuberculosis do not mimic the pathology of the disease in humans whereas tuberculosis in cattle and humans is more similar. In addition, due to the large size and large blood volume of cattle, immune cells can quite easily be obtained from various tissues, making cattle attractive models for studying the cellular effects of lectin–microbe interactions. Investigation of the cow immune response is also important for understanding potential zoonotic diseases.

Screening of large glycan arrays has been instrumental in determining specificity of lectins from various mammalian species for mammalian oligosaccharides (6–8). However, the lower availability of pure, well-characterized glycans from microorganisms is a limitation for using glycan arrays to study lectin–pathogen interactions. Arrays of glycans purified from natural sources or chemically synthesized provide one way to probe these interactions (9–11). Unfortunately, the vast majority of glycans found on bacteria, fungi, viruses, and parasites are not available in this format.

Lectin arrays have previously been constructed using plant and bacterial lectins for the purpose of deducing the structures of glycans present in samples such as glycoproteins and performing other types of glycomic analysis (12, 13). As an alternative approach, a mammalian lectin array provides the opportunity to screen many different lectins simultaneously with labeled, intact microorganisms to develop a profile in which receptors interact with each organism. Use of a lectin array constructed with bovine C-type lectins provides a rapid way to perform comparative analysis of specificity of C-type lectins and inform future studies to determine the roles of different lectins in the immune system and potentially to develop carbohydrate-based vaccines.

A model mammalian lectin array has been developed using CRDs from bovine C-type lectins modified with single-site biotin tags. The array has been used to reveal novel and unexpected interactions between bacteria and the receptors of the innate immune system.

## Results

### Identification of bovine glycan-binding receptors that contain C-type CRDs

Bovine genomic sequences in the National Center for Biotechnology Information databases were screened for annotated instances of the C-type CRD motif. To distinguish sugar-binding proteins from C-type lectin-like domains, the presence of amino acids at positions for ligating to the conserved Ca^2+^ was used to sort the annotated sequences (14). Based on these criteria, 32 potential sugar-binding proteins were identified. Most of these proteins could be identified as orthologs of human and murine proteins. The names assigned in the National Center for Biotechnology Information database accurately reflect the fact that the proteins share the same overall domain organization as the corresponding human orthologs, and the sequences of the CRDs are largely conserved (1). Most receptors found in the immune system of humans, including mincle, the receptor for trehalose dimycolate on mycobacteria, and dectin-1, which binds to β-glucans on fungi, are well-conserved in cows (15, 16). However, there is a single form of DC-SIGN in cows, compared with two in humans, and several members of the collectin subfamily, including conglutinin, CL-43, and CL-46, which appear to be ruminant-specific sugar-binding receptors (17, 18).

**Figure 1.**
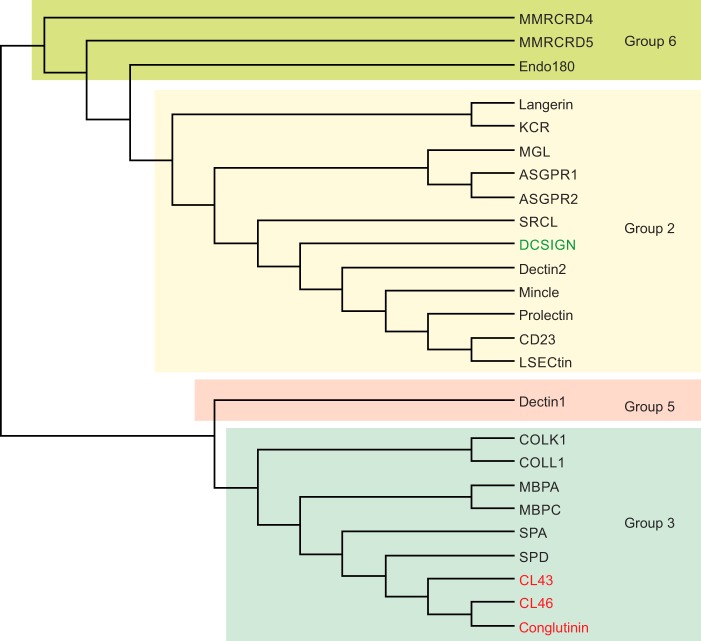
**Cow C-type CRDs.** Dendrogram shows a comparison of the CRD sequences for the CRDs from cow C-type lectins included in the lectin array. The aligned sequences are shown in Fig. S1. The lectin groups correspond to the human classification RRID:SCR_018122 (1). Only a single form of DC-SIGN (*green*) is found in cows, rather than the multiple related genes in humans and mice. Several collectins are ruminant-specific (*red*).

For the purposes of producing a prototype mammalian lectin array for analyzing interactions with microbes, CRDs from the 23 proteins highlighted in Fig. 1 were selected, based on their expression on cells of the innate immune system and exposure to the blood compartment. The cow ortholog of dectin-1 was also included as it is the only protein in which a domain with the C-type lectin fold has been shown to have sugar-binding activity despite lacking a canonical Ca^2+^-binding site (19). A mutant version of this protein, which is linked to Johne's disease, was also included for comparison (20). The resulting catalog of C-type CRD sequences is given in Fig. S1.

### Strategy for development of lectin array

**Figure 2.**
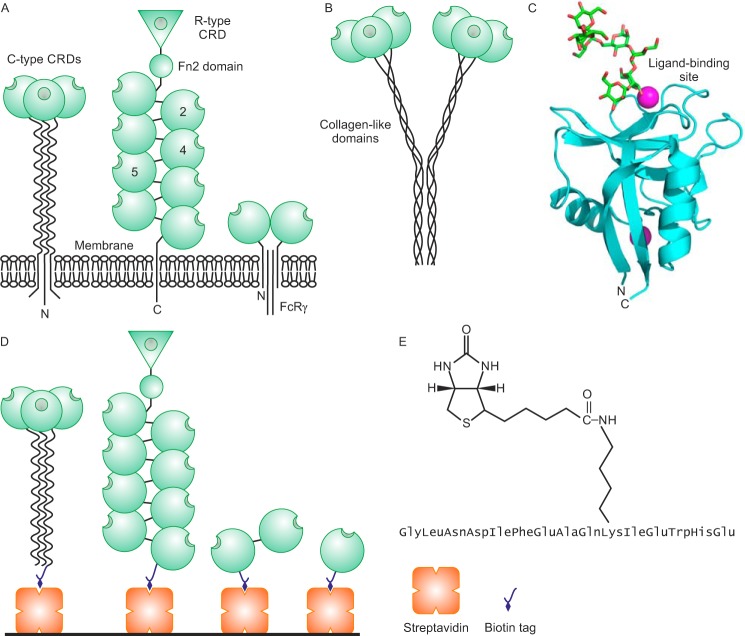
**Lectin organization and immobilization strategy.** *A*, domain organization of membrane-bound receptors containing C-type CRDs. Many of the receptors are type 2 transmembrane proteins that often assemble into oligomers (*left*). The mannose receptor and Endo180 contain multiple C-type lectin-like domains, only some of which are involved in binding sugars (*middle*). Other receptors interact with other types of polypeptides, such as the FcRγ subunit (*right*). *B*, arrangement of domains in collectins. *C*, C-type CRD from dectin-2 illustrating the location of the N and C termini opposite to the ligand-binding site. Prepared from Protein Data Bank code 5VYB using PyMOL. *D*, arrangement of lectin fragments on streptavidin-coated wells. In addition to the fragments employed in the initial test array described here, the extracellular portion of the mannose receptor expressed with a C-terminal biotinylation sequence and treated with biotin ligase *in vitro* is also depicted. *E*, sequence of biotinylation tag showing site of attachment of biotin to a lysine side chain.

Construction of the lectin array was based on use of biotinylation tags to display the CRDs in defined orientations on streptavidin-coated wells. Biotinylation was achieved by appending a 13-amino acid biotinylation sequence at one end of the CRD (21). A lysine residue in this sequence is a target for biotin ligase, which can be co-expressed with receptor fragments in *Escherichia coli* for biotinylation *in vivo* (22). Alternatively, the purified enzyme can be used to treat fragments *in vitro*. The arrangements of different C-type lectins are summarized in Fig. 2*A*, along with different ways in which N- and C-terminal biotinylation sequences have been used for immobilization. Because both ends of the polypeptide project from the face of the CRD opposite to the sugar-binding site, immobilization through tags attached to the ends of the CRDs orients the sugar-binding site projecting away from the surface of the wells. In a few cases, additional fragments larger than single CRDs have been included. The mannose receptor contains eight C-type lectin-like domains (23). CRD 4 has been shown to be the main site of sugar binding, but a pair of domains containing CRDs 4 and 5 has enhanced sugar-binding activity, so this two-domain fragment is also included in the array. As a further test case, attachment of an N-terminal biotin tag to the full extracellular portion of the Kupffer cell receptor mimics the orientation of the trimeric receptor at the cell surface (24).

Most of the tagged CRD fragments were prepared by expression in *E. coli*, followed by solubilization of inclusion bodies in guanidine and renaturation by dialysis and purification by affinity chromatography on immobilized sugars following protocols developed for many of these proteins from other species. This approach ensures that the proteins are correctly folded and have sugar-binding activity. Dectin-1 was purified by ion-exchange chromatography. Ca^2+^-independent sugar-binding activity was confirmed by immobilization in streptavidin-coated cells and probing with fluorescein-conjugated zymosan from *Saccharomyces cerevisiae* as described below. Collectin ColL1 was also purified by conventional chromatography. SDS-PAGE in the presence and absence of reducing agents indicated that disulfide bonds were formed as expected. However, no suitable sugar-containing target ligands have been identified, so it was impossible to prove unambiguously that it was correctly folded.

**Figure 3.**
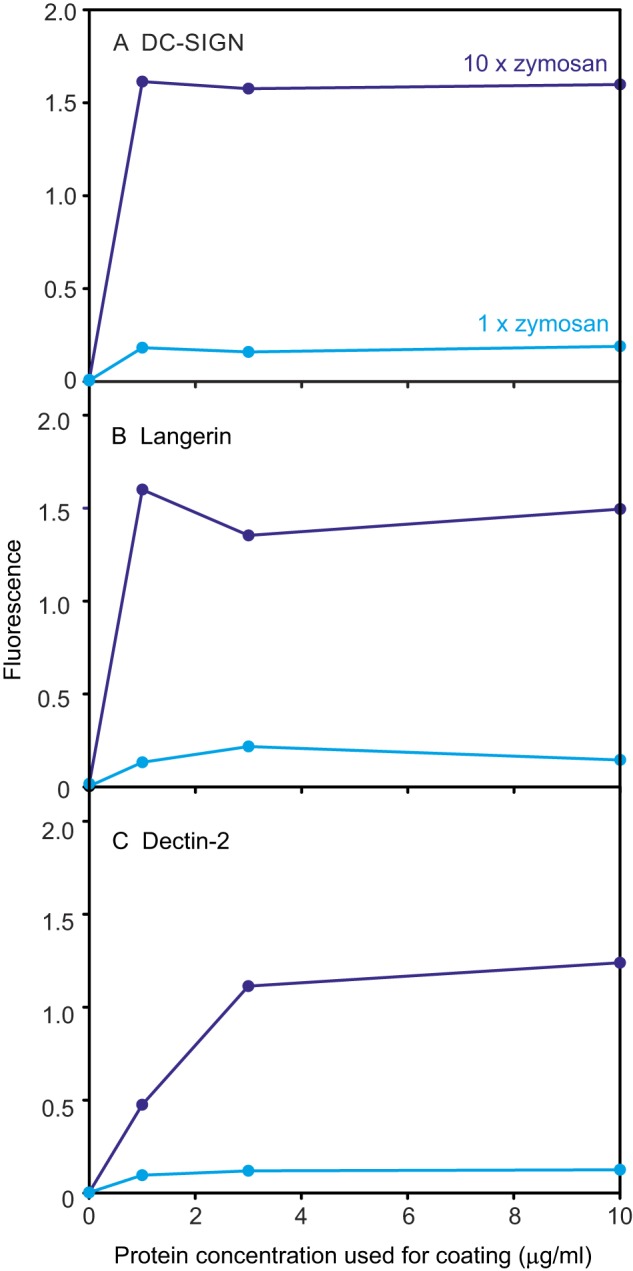
**Demonstration of saturation of streptavidin-coated wells with biotin-tagged receptors.** Examples of screening with zymosan at 5 × 10^6^ particles/ml for three receptors are shown.

For each CRD preparation, a series of different concentrations were tested in the streptavidin-coated wells. Probing with fluorescein-conjugated zymosan was used to determine what concentration of CRD was required to ensure saturation of the streptavidin in the wells. Examples of the results for DC-SIGN, dectin-2, and langerin are shown in Fig. 3. Based on these results, aliquots of each receptor preparation suitable for coating four wells were lyophilized. For the proteins that did not bind zymosan, collectin ColL1, SP-A, and mincle, saturation coating of the wells was confirmed by elution of the coated protein from the wells with SDS and analysis by SDS-PAGE. Arrays were then generated by dissolving the panel of aliquots and coating the wells in parallel.

### Validation of array using yeast zymosan

**Figure 4.**
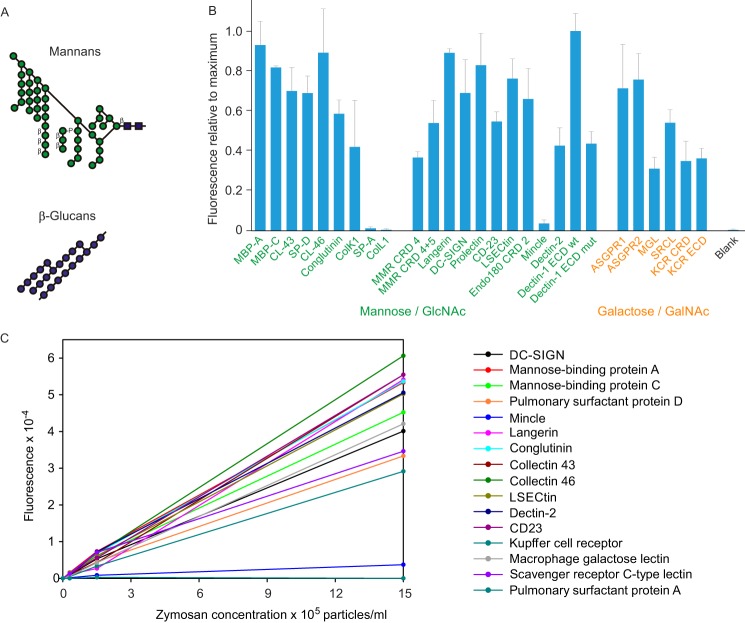
**Validation of lectin array using yeast zymosan.** *A*, structures of polysaccharides in zymosan. *B*, screening of lectin array with FITC-labeled zymosan extract from *S. cerevisiae*. Labeled zymosan at a concentration of 5 × 10^6^ particles/ml was used in the screening. The results are plotted relative to the maximal value of 215,000 fluorescence units for binding to dectin-1. In this and subsequent figures, *error bars* represent standard deviations for duplicate samples. *C*, binding as a function of zymosan concentration. Zymosan was screened against a receptor panel at three different concentrations.

Initial tests of the array were performed using zymosan particles derived from *S. cerevisiae* because this preparation contains multiple types of sugar structures and binds to most of the C-type CRDs (Fig. 4*A*). Screening of the prototype array with zymosan confirms this broad spectrum of binding (Fig. 4*B*). The lectins have been grouped based on their primary monosaccharide-binding properties. Interaction with the mannose-binding CRDs is not surprising, given the abundance of mannan structures on the surface of yeast. Interaction of zymosan with galactose-binding receptors has been previously reported and presumably reflects the presence of galactose-containing glycans (25).

When the array screening was performed at multiple different concentrations, the absolute intensity changed but the overall pattern remained the same. Analysis of binding across a range of concentrations confirmed that for most receptors the fluorescence observed was linearly related to the input concentrations of zymosan (Fig. 4*C*). This relationship indicates that the ligands were below saturating concentrations and makes the relative results broadly comparable regardless of concentration.

### Strain-dependent binding of E. coli to different C-type lectins

Laboratory strains of *E. coli* were used to test methods for preparing bacterial samples to screen against the lectin array. Initially, labeling of cells of strain BL21(DE3) by treatment with the membrane-permeable derivative of fluorescein, carboxyfluorescein diacetate succinimidyl ester (CFDA-SE), was investigated (26). Compared with direct surface labeling with fluorescein isothiocyanate (FITC), this reagent primarily reacts with amino groups inside the cells, increasing the intensity of labeling and also reducing the potential for modification of the surface properties of the bacteria.

**Figure 5.**
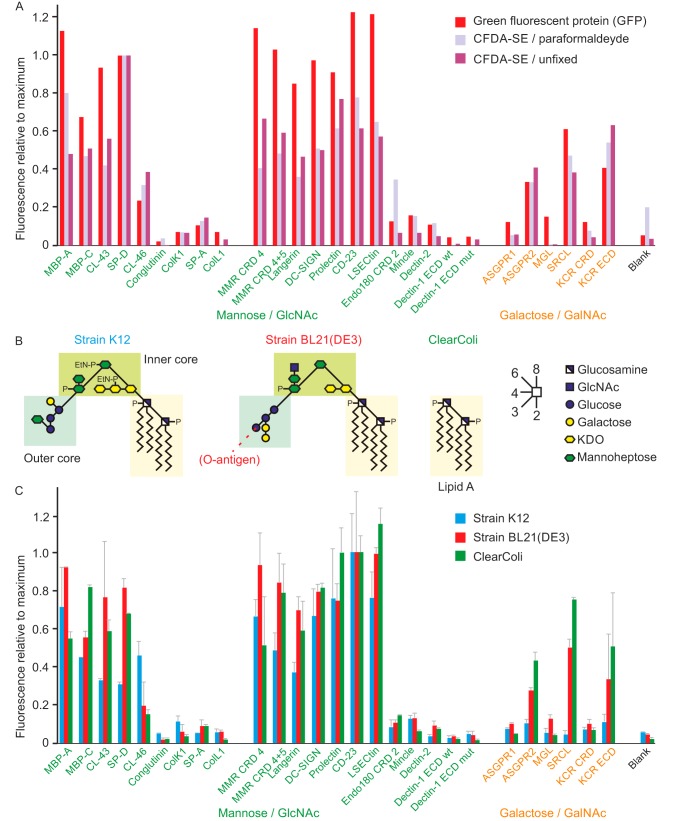
**Testing of lectin array with laboratory *E. coli* strains.** *A*, comparison of different methods for preparing bacterial strain BL21(DE3) prior to screening of the array. Cells expressing GFP and fixed with paraformaldehyde were screened at 1 × 10^8^ cells/ml, and cells labeled with CFDA-SE followed by paraformaldehyde fixation or used directly were screened at 3 × 10^8^ cells/ml. For comparison, results for all three samples were normalized to the SP-D result, which was the highest value for both of the CFDA-SE samples. The values were 40,400, 16,400, and 39,000 fluorescence units, respectively. *B*, summary of structures of LPS from three laboratory strains of *E. coli. C*, binding of different strains of *E. coli* to the lectin array. Strain K12 cells were labeled with FITC (1.5 × 10^7^ cells/ml), and BL21(DE3) (5 × 10^7^ cells/ml) and the ClearColi derivative of BL21(DE3) (3 × 10^8^ cells/ml) were expressing GFP. All results were normalized to the signals for CD23, which was the strongest signal for K12 and BL21(DE3). The signals were 56,000, 49,700, and 46,200 fluorescence units for K12, BL21(DE3), and BL21(DE3) ClearColi, respectively.

Screening of the lectin array directly with live bacteria or following paraformaldehyde fixation of the bacteria gave similar results (Fig. 5*A*). Compared with zymosan, a more restricted set of receptors gave signals for the bacterial samples. Many of the membrane receptors and serum collectins that bind mannose-containing glycans bound well to the bacteria, but there are notable exceptions, such as conglutinin, Endo180, dectin-2, and dectin-1, which bound well to zymosan but not to the bacteria. Generally, lower signals were observed for galactose-specific receptors, but again there was selective binding: to one of the subunits of the asialoglycoprotein receptor, to the scavenger receptor C-type lectin, and to the Kupffer cell receptor.

Bacteria could also be labeled with fluorescent proteins (27). Comparing the results for strain BL21(DE3) expressing green fluorescent protein (GFP) to the chemically-labeled cells shows very good concordance of results (Fig. 5*A*). Compared with chemical labeling, expressing fluorescent proteins can be more species-restricted, because appropriate promotors need to be used for expression. However, many bacteria expressing fluorescent proteins have been developed for use in endocytosis assays, for example, and the results shown here indicate that such samples can be adapted for use in screening the array (27).

Lipopolysaccharide (LPS) on the surface of the outer membrane of *E. coli* strain BL21(DE3) contains inner and outer core structures, but no O-antigen extensions (Fig. 5*B*) (28). Residues at the nonreducing termini of these glycans are potential targets for the lectins that were observed to bind the bacteria. The presence of some terminal galactose residues is consistent with the observed binding of galactose-specific lectins, and the presence of glucose and GlcNAc residues might account for binding of receptors that bind mannose-related sugars. However, robust binding is observed for ClearColi BL21(DE3) cells, a derivative of strain BL21(DE3) that has only the lipid A portion of LPS (Fig. 5*C*). The close similarity in the binding patterns for BL21(DE3) and ClearColi BL21(DE3) cells suggests that there are other targets besides those on LPS.

A further comparison was made with *E. coli* strain K12, which also lacks an O-antigen and has an LPS outer core structure distinct from the LPS on BL21(DE3) (29). Compared with strain BL21(DE3), broadly similar binding to lectins that bind mannose-type sugars was observed, but there was little binding to any of the galactose-specific receptors, even though there is a galactose residue in the LPS core glycan (Fig. 5*C*). These results are consistent with the suggestion that a significant component of the binding to several of the receptors is LPS-independent, probably reflecting the presence of additional glycosylated structures on the surface of the outer membrane.

**Figure 6.**
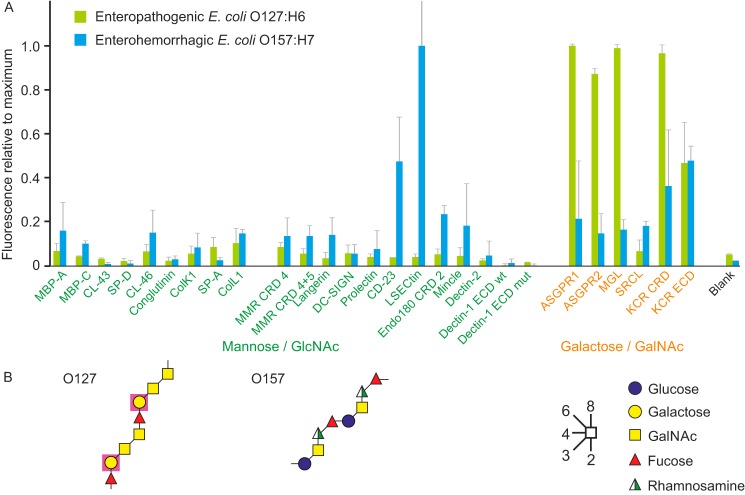
**Comparison of enteropathogenic and enterohemorrhagic *E. coli* strains binding to the lectin array.** *A*, bacteria expressing GFP were grown to stationary phase and fixed with paraformaldehyde. Enteropathogenic *E. coli* strain E2348/69 (O127:H6) was screened at 7 × 10^8^ cells/ml and enterohemorrhagic *E. coli* strain EDL933 (O157:H7) was screened at 5 × 10^8^ cells/ml. Results were normalized to the values for ASGPR1 (41,400 fluorescence units) and LSECtin (51,800 fluorescence units), respectively. *B*, structures of two repeat units from the O127 and O157 outer polysaccharides of LPS are shown. *Purple shading* is used to highlight galactose residues that have free 3- and 4-OH groups in the O127 polysaccharide and would be potential targets for galactose-binding C-type CRDs.

Two examples of pathogenic *E. coli* serotypes that have been isolated from human and cattle were also tested. Strain E2348/69 (O127:H6), a commonly used prototype enteropathogenic strain, showed strong binding to almost all of the galactose-specific receptors, with the exception of the scavenger receptor C-type lectin, which has a narrow specificity for galactose in the context of the Lewis^x^ trisaccharide (Fig. 6*A*). The strong binding correlates with the presence of galactose in the repeat unit of the O127 polysaccharide (Fig. 6*B*) (30). The galactose residue present in each copy of the backbone repeat would be a target for the galactose-binding C-type lectins, because it retains free 3- and 4-OH groups. The adaptive immune response to most bacteria is dominated by the O-antigens, but these results suggest that some O-antigens could also be targets for receptors of the innate immune system.

In contrast, enterohemorrhagic *E. coli* strain EDL933 (O157:H7) binds very few receptors on the array, although CD23 and LSECtin show consistent signals (Fig. 6*A*). There is no obvious target for binding to these receptors in the O-antigen structure (Fig. 6*B*) (31). If found at the nonreducing termini of the polysaccharides, the constituent sugars GalNAc, glucose, and fucose would be potential targets for receptors. However, the absence of binding to most of the receptors indicates that there are few such terminal sugars exposed on the O157:H7 strain. The finding that terminal sugars are not common targets in the O157:H7 strain, compared with the strong binding to the O127:H6 strain to multiple receptors, suggests that binding to O-antigens is likely to be dominated by internal sugars that have appropriately exposed OH groups. The poor binding of the O157:H7 strain to most receptors also demonstrates that expression of O-antigens lacking target residues provides a way to escape detection by the innate immune system. Many common modifications, such as methylation and acetylation, block the 3- and 4-OH groups and would thus prevent binding (32).

One possible target for binding across multiple bacterial strains is the enterobacterial common antigen, which can appear in a linear, lipid-linked form on the surface of the outer membrane (33). Terminal GlcNAc residues in the trisaccharide repeat might be potential targets for some of the receptors. The observation that the O157:H7 strain does not bind to most of the receptors that would be expected to interact with GlcNAc suggests that this is not a common target, although the extent to which the antigen is surface-expressed in different strains is not fully characterized.

### Distinct binding of capsulated and unencapsulated Klebsiella pneumoniae

**Figure 7.**
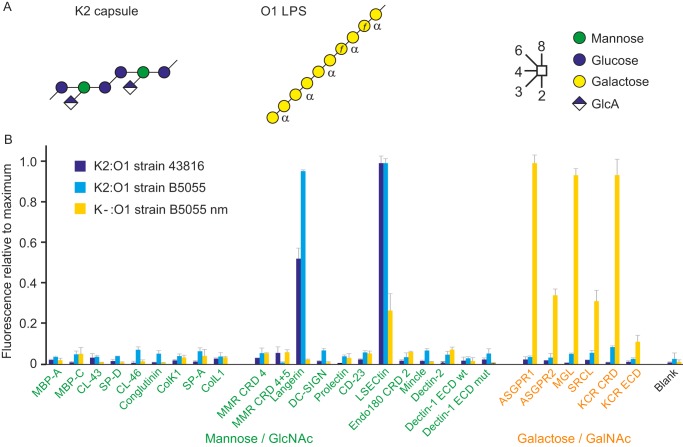
**Effect of capsule on interaction of *K. pneumoniae* with lectins on the array.** *A*, summary of structures of the K2 capsule and the O1 outer polysaccharide structure of *K. pneumoniae. B*, lectin array was screened with two strains of *K. pneumoniae* with the K2:O1 serotype, strains 43816 and B5055, as well a mutant derived from the B5055 strain in which the *wzb/c* gene is inactivated to prevent capsule formation. Cells expressing GFP were grown to stationary phase and screened against the array at concentrations of 9 × 10^8^, 4 × 10^8^, and 7 × 10^8^ cells/ml, respectively. Results were normalized to the signals for LSECtin for the WT strains (155,000 and 37,300 fluorescence units) and ASGPR1 for the mutant strain (796,000 fluorescence units).

WT K2:O1 *K. pneumoniae*, a hyperviscous pathogen for human and cattle, is coated with a capsule consisting of a glucose- and mannose-containing backbone, with glucuronic acid side branches (Fig. 7*A*) (34). Screening against the lectin array revealed binding to two receptors, langerin and LSECtin. The fact that the capsule is the target for binding to these receptors was demonstrated by screening a mutant strain that lacks the capsule (Fig. 7*B*). In contrast to the WT strain, the mutant binds to almost all of the galactose-specific receptors and not to langerin and LSECtin. The galactose-dependent binding probably reflects exposure of the LPS glycans that contain galactopyranose residues. However, in this case it would have to be terminal residues that bind, because the linkages in the backbone would preclude interaction of internal residues with any of the receptors (Fig. 7*A*) (35). Comparison of encapsulated and unencapsulated strains indicates that the outer polysaccharide shields the LPS from receptors and dominates the spectrum of interactions with glycan-binding receptors.

The 1–4 and 1–3 linkages in the backbone of the capsular polysaccharide would block binding to the 3- and 4-OH groups of these residues, making them unavailable to interact with langerin and LSECtin (Fig. 7*A*). However, in the absence of binding to any of the other receptors that recognize these sugars, it seems unlikely that interactions with nonreducing terminal sugar residues could account for the observed binding. Langerin does bind negatively-charged sulfated sugar residues (36), but testing of simple glucuronic acid methyl glycoside in a solid-phase binding competition assay showed only very weak interaction (data not shown). It remains possible that the glucuronic acid forms part of a more complex binding epitope.

### Receptors that bind Gram-positive Staphylococcus aureus

**Figure 8.**
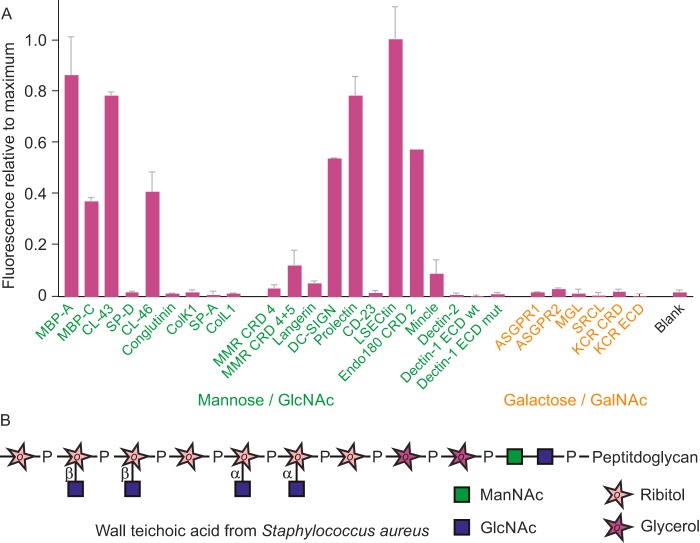
***S. aureus* binding to lectin array.** *A*, heat-killed cells of the Wood 46 strain of *S. aureus* labeled with FITC were used to screen the array at a concentration of 1.25 × 10^8^ cells/ml. Results were normalized to the signal of 109,000 fluorescence units, which was obtained for LSECtin. *B*, schematic diagram of wall teichoic acid from *S. aureus*, showing relative location of α- and β-linked GlcNAc residues. Only β-linked residues are present in this strain.

Probing of the lectin array with *S. aureus*, a Gram-positive bacterium that has been isolated from human and cattle, revealed binding to a more limited set of receptors compared with *E. coli* (Fig. 8*A*). The Wood 46 strain of *S. aureus* lacks protein A and is *tarS*^+^/*tarM*^−^, leading to expression of β- but not α-linked surface GlcNAc (37). Similar results were obtained with independent preparations of labeled bacteria. The main receptor that gives a relatively strong signal with *S. aureus* but not with *E. coli* is CRD2 of Endo180. Although this receptor is classified with the mannose-type binding sites, it shows a very strong preference for GlcNAc compared with other monosaccharides (38). It is likely that the binding observed correlates with the ability of this CRD to bind to β-linked GlcNAc that decorates the wall teichoic acid (Fig. 8*B*) (39). These residues would also be targets for binding other receptors such as DC-SIGN. However, it is striking that the mannose receptor and langerin show little or no binding despite their ability to interact with GlcNAc as a monosaccharide (40). In contrast, LSECtin, a receptor previously associated with binding GlcNAcβ1–2Man structures on truncated glycans of viruses (41), gives a strong signal. Although the LSECtin result would appear to be consistent with GlcNAc binding on *S. aureus*, the primary binding residue observed in viral glycans is the mannose residue. The array data suggest that there are important aspects to the specificity of some of the receptors that remain to be investigated both to explain why receptors such as langerin do not bind GlcNAc in the context of the teichoic acid and to identify novel modes of ligand binding in other cases such as LSECtin.

As with the membrane receptors, there is striking specificity in the binding to a subset of the collectins: the four serum collectins MBP-A, MBP-C, CL-43, and CL-46 give strong signals, but conglutinin, another serum protein, does not. The binding sites in MBP-A and MBP-C are relatively unencumbered, so they interact equally well with mannose and GlcNAc (42). However, the monosaccharide-binding specificity of both conglutinin and CL-46 is for GlcNAc (43). The difference in their interaction with *S. aureus* suggests that, as in the case of the mannose receptor and langerin, the specific structural context of the GlcNAc linked to teichoic acid might limit the interaction with conglutinin. It is more difficult to explain the observed strong interaction with CL-43, which at the monosaccharide level binds best to mannose. The results suggest that there may be nuances in the ligand-binding specificities of the various collectins that have not previously been recognized or that other ligands are present on the surface of these bacteria.

### Receptor for Mycobacterium bovis

**Figure 9.**
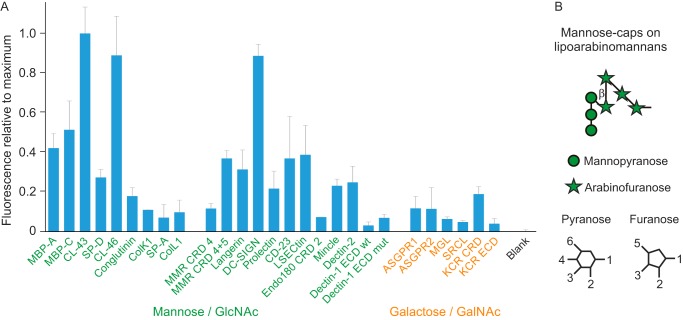
***M. bovis* binding to lectin array.** *A, M. bovis* bacillus Calmette-Guerin expressing yellow fluorescent protein growing in log phase was further labeled with CFDA-SE, fixed with paraformaldehyde, and used to screen the lectin array at a concentration of 4 × 10^8^ cells/ml. Results were normalized to the value for binding to CL-43, which was 30,000 fluorescence units. *B*, structure of mannose caps on lipo-arabinomannan in the outer membrane of *M. bovis*.

Mycobacteria have a complex membrane structure decorated with novel classes of glycans compared with the other bacteria investigated here, and there are corresponding differences in the ability of different receptors to interact with labeled *M. bovis* (Fig. 9*A*) (44). Within the collectin family, for instance, the strongest signals are observed for CL-43 and CL-46, which are not observed for any of the other bacteria tested. Although much of the binding can be explained by interaction with mannose caps on the lipoarabinomannans of the outer membrane, the results suggest that the collectins may be particularly suited to binding these α1–2–linked mannose structures (Fig. 9*B*). In a similar way, there is some binding to dectin-2, for which Manα1–2Man is a preferred ligand (45, 46). Dectin-2 binds to zymosan, which contains such structures, but not to the other bacteria tested on the array. Most of the other receptors for mannose-type ligands also bind the mycobacteria, including DC-SIGN and the mannose receptor, which have been suggested to be the major routes for entry into cells (47). However, a unique feature of mycobacteria binding to the array is that there is some interaction with mincle, a receptor that does not bind to the Gram-negative bacteria. Although it is likely that the mannose-binding receptors mediate entry into cells, mincle binding to trehalose dimycolate in the bacterial outer membrane is an important signaling mechanism in macrophages (48), and the observation that binding is sufficient to tether the mycobacteria to the immobilized receptor confirms that this ligand is accessible on the surface of the intact mycobacteria.

## Discussion

The design of the lectin array described here includes a number of important technical features associated with the use of biotin tags for immobilization. A key point is the use of simple bacterial expression systems that are easy to generate and give high yields. Biotin tagging takes place co-translationally in the expression system. The activity of the proteins is confirmed by affinity chromatography on sugar ligands. Using lectins with confirmed activity gives confidence in negative as well as positive results. The streptavidin layer creates a soft protein surface. Because the tag is on the opposite side of the protein from the sugar-binding site, it does not interfere with binding and orientation of the binding sites away from the layer is ensured. Including additional domains beyond the CRD, particularly coiled-coil neck domains, allows formation of oligomers in which the CRDs are held in the native orientation. The biotin-tagged proteins can also be used in follow-up experiments, such as using fluorescently-labeled streptavidin to create probes for investigating the nature of ligands in microbes that are found to bind to specific receptors.

An advantage of the lectin array is the ability to provide an unbiased search for receptors that bind to a particular microorganism, independent of any knowledge of what sugar structures are present on the microbe. This feature allows screening with organisms that have not been subjected to extensive glycomic analysis, but it also has the potential to reveal unrecognized types of glycosylation even in relatively well-characterized microbes. Use of minimally-modified microorganisms to screen the array demonstrates accessibility of glycan ligands in the context of intact microorganisms. A further advantage of the array approach is the ability to compare binding of a bacterium or other microorganism to multiple potential receptors in one assay. The use of a species-specific array also makes it possible to perform cross-species studies to identify differences in binding of microbes between different host species. Such results can potentially lead to the discovery and understanding of zoonosis. Given that many C-type lectins are expressed on cells that sample mucosal surfaces, this knowledge can be used to identify new intervention strategies to reduce pathogen pressure on surfaces, to prevent entry of pathogens, or for the development of carbohydrate-based vaccines.

**Figure 10.**
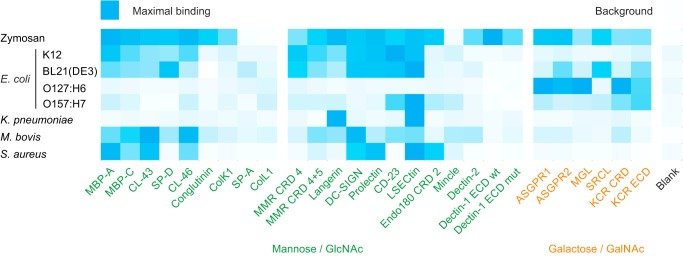
**Comparative heatmap for zymosan and bacteria binding to lectin array.** Binding of laboratory and pathogenic strains of *E. coli,* as well as *K. pneumoniae*, *M. bovis,* and *S. aureus,* are compared with zymosan results using a heatmap. Results for each organism are normalized to the maximum value for that organism as indicated in legends to Figs. 4–9.

Interesting outcomes from these initial applications of the array, summarized in the heatmap in Fig. 10, include unexpected aspects of microbial glycosylation and insights into novel binding properties of some of the receptors. The binding of laboratory strains of *E. coli* to multiple receptors, apparently independent of the presence of even core sugars of LPS, indicates that there are additional types of glycosylation exposed on these cells. Potential targets include novel types of protein-linked glycosylation (49). The O-antigens found on the LPS of pathogenic strains of *E. coli* are strain-specific, so they can bind to different receptors depending on the sugar composition of a given strain. These structures dominate the antibody response to different strains, but the results with the array highlight the fact that different O-antigens will also lead to different interactions with the innate immune system. In principle, innate immune recognition of common structures allows immediate response to whole classes of bacteria in a strain-independent manner. However, comparing the results for laboratory strains in Fig. 5 with the pathogenic strains in Fig. 6 reveals very different patterns of interactions. Common structures on the surface of laboratory strains, which lack O-antigens, interact with a range of receptors, but these interactions are dwarfed by the dominant strain-specific interactions when O-antigens are present.

Contrasting the interactions of different naturally-occurring strains of bacteria with lectins on the array provides insights into the molecular nature of potential ligands, such as the O-antigens. The analysis of *Klebsiella* mutants demonstrates the power of combining the array with bacterial mutants as an additional way to define potential ligands. In this instance, it was possible to distinguish interactions with the capsule and LPS. In other cases, the biotin-tagged CRDs can be used as probes to identify target molecules, for example by using streptavidin complexes to probe blots of glycoproteins and glycolipids extracted from the bacteria.

The results with the array also reveal novel aspects of receptor specificity, negative as well as positive. For example, there are striking differences in the interactions of DC-SIGN on the one hand and the mannose receptor and langerin on the other, even though these receptors bind a similar spectrum of monosaccharides. The specific *S. aureus* strain used in this study shows particularly strong preferential binding to DC-SIGN, but *M. bovis* also gives a stronger signal with DC-SIGN. Although all of these receptors can bind to the putative GlcNAc target on *S. aureus* and to terminal Manα1–2Man structures that appear in the caps of mycobacterial lipoarabinomannans, the context in which these binding epitopes are presented seems to differentially affect their interactions with receptors.

Several of the receptors on the array show interactions that would not have been expected based on what is known about their interactions with defined glycan ligands. For example, on mammalian glycan arrays the scavenger receptor C-type lectin binds very selectively to Lewis^x^-containing oligosaccharides (50), but it also seems to interact with the exposed, galactose-containing LPS of the *Klebsiella* capsule mutant. Langerin and LSECtin bind especially well to sugars present in the capsule of *Klebsiella*, even though the capsule structure does not contain any obvious targets based on what is known about the specificities of these two receptors. On glycan arrays, LSECtin binds to oligosaccharides containing the disaccharide GlcNAcβ1–2Man (41), but this narrow specificity does not explain its interactions with enterohemorrhagic *E. coli*, *Klebsiella,* and *S. aureus*. These results suggests that further screening with a broader range of potential oligosaccharide ligands will reveal novel types of ligands.

The array also highlights differences in the ways that the serum collectins interact with microorganisms. The serum mannose-binding proteins and the ruminant-specific proteins CL-43 and CL-46 all interact with mycobacteria, although the additional ruminant-specific collectin, conglutinin, does not. The proliferation of collectins in cows might be related to the evolutionary history of infection with mycobacteria. The collectins also interact particularly well with Gram-positive *S. aureus*, but poorly with the Gram-negative strains tested. This selectivity correlates with the fact that infections associated with deficiency of human mannose-binding proteins are often caused by Gram-positive species (51). These results suggest that innate immune responses have evolved to target specific classes of potential pathogens, an idea that is also supported by the observations that both dectin-1 and dectin-2 appear to interact selectively with fungi rather than bacterial targets. The ability to compare WT and naturally-occurring mutant forms of receptors such as dectin-1 on the array also provides a way of investigating how polymorphisms that affect the sequences of receptors may change the way that they interact with pathogens. In some cases, such changes may explain disease phenotypes (20).

Future developments of the lectin array can include addition of other classes of glycan-binding proteins to the array. Other lectin groups in which multiple members have potential roles in recognition of microorganisms include the siglecs and the galectins. The availability of bacterial expression systems for the sugar-binding domains of these proteins will facilitate addition of biotin tags using the approach described here for co-expression with biotin ligase (52, 53). However, in cases where the use of mammalian expression systems is preferable, it is also possible to attach the biotin to the appended target sequence after isolation of the protein. Such an approach has been employed for the multidomain extracellular portion of the mannose receptor, which has been expressed in secreted form in Chinese hamster ovary cells, purified by affinity chromatography, and treated with biotin ligase before immobilization (data not shown). The approach demonstrated here for the cow lectins can also be readily duplicated for receptors from other organisms, including humans and mice. In addition, further categories of microorganisms, including viruses and fungi, can be screened against the array.

## Experimental procedures

### Database screening

Genes encoding proteins that contain potential CRDs were identified by screening the National Center for Biotechnology Information gene database using *Bos taurus* and CLECT as keywords. The DNA sequence of the region encoding a potential CRD was extracted from each file in reference assembly version ARS-UCD1.2. All of the sequences containing amino acid residues at appropriate positions to form the conserved Ca^2+^-binding site were identified based on the database annotation and comparison with the human and mouse sequences. CRDs selected for inclusion on the initial array were subjected to cluster analysis with ClustalW, and the results were used to generate a cladogram using Dendroscope 3 (54, 55). The aligned sequences (Fig. S1), along with previous instances in which human orthologs have been expressed, were used to define regions of each protein to be expressed.

### Expression systems

Fragments for making expression vectors were generated by PCR amplification using primers that encode the biotin tag at the 3′ or 5′ end and restriction sites to allow movement into the expression vector. cDNAs were amplified using Advantage2 polymerase (Takara) (Figs. S2–S28). cDNA libraries prepared from various tissues as indicated in Table 1 were obtained from Zyagen through AMSBio. Amplified fragments were cloned into vector pCRIITopo (Life Technologies, Inc.), and the sequences were confirmed. For Endo180, a synthetic version of CRD 2 predicted from the gene sequence was synthesized by GeneArt (Fig. S19). Restriction fragments were transferred to the pT5T expression vector (56).

**Table 1:** **Biotin-tagged C-type lectins displayed on lectin array** cDNA libraries used for PCR amplification, methods for protein renaturation, and resins used for purification are indicated for each protein.

Abbreviation	Protein	Gene	Library	Renaturation	Resin
MBP-A	Mannose-binding protein A	*MBL1*	Liver	Direct	Mannose
MBP-C	Mannose-binding protein C	*MBL2*	Liver	Direct	Mannose
CL-43	Collectin 43	*CL43*	Liver	Direct	Mannose
SP-D	Surfactant protein D	*SFTPD*	Lung	Direct	Maltose
CL-46	Collectin 46	*CL46*	Liver	Direct	GlcNAc
Conglutinin	Conglutinin	*CGN1*	Liver	Direct	GlcNAc
ColK1	Collectin K1	*COLEC11*	Liver	Direct	Mannose
SP-A	Surfactant protein A	*SFTPA*	Lung	Lyophilized	Mannose
ColL1	Collectin L1	*COLEC12*	Liver	Lyophilized	Mono Q
MMR CRD 4	Mannose receptor CRD 4	*MRC1*	Liver	Direct	Mannose
MMR CRD 4 + 5	Mannose receptor CRD 4 + 5	*MRC1*	Liver	Dilution	Mannose
Langerin	Langerin	*CD207*	Lung	Direct	Mannose
DC-SIGN	DC-SIGN	*CD209*	Spleen	Direct	Mannose
Prolectin	Prolectin	*CLEC17A*	Spleen	Direct	Mannose
CD23	CD23	*FCER2*	Spleen	Direct	Mannose
LSECtin	LSECtin	*CLEC4G*	Liver	Lyophilized	Mannose
Endo180 CRD 2	Endo180	*MRC2*	-	Direct	GlcNAc
Mincle	Mincle	*CLEC4E*	Liver	Direct	Trehalose
Dectin-2	Dectin-2	*CELC6A*	Lung	Triton	Mannose
Dectin-1 ECD WT	Dectin-1 wildtype	*CLEC7A*	Lung	Triton	Mono Q
Dectin-1 ECD mut	Dectin-1 mutant	*CLEC7A*	Lung	Triton	Mono Q
ASGPR1	Asialoglycoprotein receptor 1	*ASGR1*	Liver	Direct	Galactose
ASGPR2	Asialoglycoprotein receptor 2	*ASGR2*	Liver	Direct	Galactose
MGL	Macrophage galactose receptor	*CLEC10A*	Lung	Dilution	Galactose
SRCL	Scavenger receptor C-type lectin	*COLEC12*	Lung	Direct	Galactose
KCR CRD	Kupffer cell receptor	*CLEC4F*	Liver	Dilution	Galactose
KCR ECD	Kupffer cell receptor trimer	*CLEC4F*	Liver	Dilution	Galactose

Biotin-tagged CRDs were expressed from the T7 promotor in plasmid pT5T in *E. coli* strain BL21(DE3) that also carried plasmid pBirA, which encodes biotin ligase (21). Bacteria were grown with shaking in Luria-Bertani medium containing 50 μg/ml ampicillin and 25 μg/ml chloramphenicol at 37 °C. Isopropyl β-d-thiogalactoside was added to a concentration of 100 mg/liter when the *A*_550_ reached 0.7, followed by a further 150-min incubation. Bacteria were harvested by centrifuging at 4000 × *g* for 15 min at 4 °C, washed with 10 mm Tris-HCl, pH 7.8, and pelleted at 10,000 × *g* for 10 min at 4 °C.

Cell pellets from 6 liters of culture in 200 ml of 10 mm Tris-Cl, pH 7.8, were sonicated four times for 1 min at full power using a Branson 250 sonicator. Inclusion bodies recovered by centrifugation for 15 min at 10,000 × *g* in a Beckman JA-14 rotor were dissolved by homogenization in 100 ml of 6 m guanidine-HCl, 100 mm Tris-Cl, pH 7.0. 2-Mercaptoethanol was added to a final concentration of 0.01%, and the mixture was incubated for 30 min at 4 °C and centrifuged for 30 min at 40,000 × *g* in a Beckman 70.1Ti rotor.

Proteins were renatured by one of three different protocols, as indicated in Table 1. In the direct dialysis protocol, the guanidine solution was dialyzed against three changes of 2 liters of renaturation buffer (0.5 m NaCl, 25 mm Tris-Cl, pH 7.8, 25 mm CaCl_2_). For renaturation with Triton detergent, 10 ml of 10% Triton X-100 was added to the guanidine solution before dialysis. In the dilution protocol, the guanidine solution was dripped into 400 ml of renaturation buffer before dialysis against three changes of 4 liters of renaturation buffer. For proteins that bound weakly to the affinity columns, material renatured by the direct protocol was further dialyzed against three changes of water, lyophilized, and resuspended in a 10-fold reduced volume of loading buffer, consisting of 0.5 m NaCl, 25 mm Tris-Cl, pH 7.8, 25 mm CaCl_2_ for SP-A and a 20-fold reduced volume of loading buffer, consisting of 0.15 m NaCl, 25 mm Tris-Cl, pH 7.8, 25 mm CaCl_2_ for LSECtin, before affinity chromatography.

In most cases, the final dialyzed samples were centrifuged for 30 min at 20,000 × *g* in a Thermo Fisher Scientific F21S-8 × 50g rotor or Beckman JA-14 rotor and applied to 10-ml affinity columns made with appropriate immobilized sugar ligands (57). Columns were washed with 10 ml of 150 mm NaCl, 25 mm Tris-Cl, pH 7.8, 25 mm CaCl_2_ and eluted with 15 1-ml aliquots of 150 mm NaCl, 25 mm Tris-Cl, pH 7.8, 2.5 mm EDTA. Fractions were examined on SDS-polyacrylamide gels containing 17.5% polyacrylamide. The SP-A protein eluted early, during the wash, so fractions were collected earlier. Wash fractions containing SP-A protein were pooled, dialyzed against water, lyophilized, resuspended in 1 ml of loading buffer containing 0.15 m NaCl, and repurified over the 10-ml mannose column.

In two instances, dectin-1 and the collectin ColL1, the CRDs could not be purified by affinity chromatography. Following renaturation by the Triton and lyophilized protocols, respectively, these proteins were dialyzed against or resuspended in 50 mm Tris-Cl, pH 7.8, and loaded onto a 1-ml Mono Q ion-exchange column, which was eluted with a gradient from 0 to 0.5 m NaCl containing 50 mm Tris-Cl, pH 7.8. Fractions containing the renatured CRDs, identified by SDS-PAGE, were pooled, dialyzed against water, and lyophilized. ColL1 and CRDs 4 + 5 of the mannose receptor were further purified by gel-filtration chromatography on a 1 × 30-cm Superdex S75 column eluted with 100 mm NaCl, 10 mm Tris-Cl, pH 7.8, 2.5 mm EDTA, and the purified protein was again detected by SDS-PAGE.

### Labeled microorganisms

Bacterial strains used are summarized in Table S1. Fluorescein-labeled preparations of zymosan from *S. cerevisiae*, *E. coli* K12, and the Wood 46 strain of *S. aureus* were obtained from Molecular Probes (Thermo Fisher Scientific). Heat-killed *S. aureus* Wood 46 cells from Invivogen suspended in 1 ml of 0.1 m sodium carbonate, pH 9.0, were treated with 100 μl of 1 mg/ml FITC in DMSO. For labeling with CFDA-SE, bacteria were washed and suspended in PBS. An aliquot (1 ml) was treated with 2 μl of 50 mm CFDA-SE in DMSO. After reaction for 6–18 h with gentle mixing at room temperature, cells were again washed twice with PBS, treated for 2 h with 5% paraformaldehyde at room temperature, and finally washed three times with TBS.

A plasmid containing a cDNA for enhanced GFP in vector pET28a, a kind gift of Neil Fairweather, was transformed into *E. coli* strains BL21(DE3) and into competent ClearColi BL21(DE3) cells obtained from Lucigen. Other *E. coli* and *K. pneumoniae* strains were transformed with plasmids expressing GFP from pACYC184 (enteropathogenic *E. coli*) or pUltra-GFP/Gn (all other strains) (58, 59). Cells were grown to stationary phase, washed in PBS, fixed for 30 min in 5% paraformaldehyde in PBS, and washed with TBS.

*M. bovis* strain bacillus Calmette-Guerin Pasteur Δ*panCD* expressing yellow fluorescent protein from promotor P_smyc_ contained vector pCB22-Turbo635-ASV-YFP. This plasmid was created by cloning the region encoding the Turbo635-ASV/msfYFP operon from plasmid pCharge-ASV-YFP into the multiple cloning site of shuttle vector pCB22, which contains both an *E. coli* and a mycobacterial origin of replication, with a hygromycin-resistance cassette and a *panCD* operon driven by the constitutive BCG_3667c promoter, followed by the multiple-cloning site (60). Mycobacteria were grown at 37 °C in Middlebrook 7H9 broth (BD Biosciences) supplemented with 0.05% Tween 80 (Sigma), 0.2% glycerol (Sigma), and 10% oleic acid/albumin/dextrose/catalase (BD Biosciences). To obtain fluorescence levels above the background threshold, the fluorescent mycobacteria were additionally labeled with CFDA-SE. Log-phase cultures were washed twice and suspended in 1 ml of PBS + 0.05% Tween 80 at a concentration of 7.0 × 10^8^ cells/ml. Bacteria were treated with 2 μl of 50 mm CFDA-SE, incubated with gentle shaking for 18 h at 37 °C, washed with TBS, fixed with 5% formaldehyde for 2 h at room temperature, and further washed with TBS.

Bacteria were quantified by counting of suitable dilutions in a Helber counting chamber, and total fluorescence of the samples was determined for an aliquot of cells diluted in PBS and applied to a 96-well plate.

### Array screening

All procedures were conducted in binding buffer containing 0.15 m NaCl, 25 mm Tris-Cl, pH 7.8, 2.5 mm CaCl_2_. Biotinylated receptor fragments were dissolved in binding buffer containing 0.1% BSA. Streptavidin-coated wells from Pierce (Thermo Fisher Scientific) were incubated overnight at 4 °C with 100-μl aliquots of these coating stocks, and the wells were washed three times with binding buffer. Labeled microorganisms suspended in binding buffer containing 0.1% BSA were added to duplicate wells in 100-μl aliquots. After incubation for 3 h at 4 °C, wells were washed three times with binding buffer and scanned directly on a Victor3 multiwell plate reader (PerkinElmer Life Sciences).

## Author contributions

S. A. F. J., D. W., A. C., M. E. T., and K. D. conceptualization; S. A. F. J., M. E. T., and K. D. formal analysis; S. A. F. J., M. E. T., and K. D. supervision; S. A. F. J., C. N., T. H., S. T. A. W., N. K. K. L., G. K. E. N., R. C.-R., Z. H., B. M., A. Hirdaramani, A. Howitt, K. F., Z. S., R. J. F., R. W., M. A., L. E., H. Z., A. Holder, N. K., A. C., M. E. T., and K. D. investigation; S. A. F. J., M. E. T., and K. D. writing-original draft; S. A. F. J., D. W., M. E. T., and K. D. project administration; S. A. F. J., C. N., T. H., S. T. A. W., N. K. K. L., G. K. E. N., R. C.-R., Z. H., B. M., A. Hirdaramani, A. Howitt, K. F., Z. S., R. J. F., R. W., M. A., L. E., H. Z., A. Holder, D. W., N. K., B. D. R., A. C., M. E. T., and K. D. writing-review and editing; D. W., M. E. T., and K. D. funding acquisition; M. E. T. and K. D. visualization.

## Supplementary Material

Supporting Information

## Footnotes

2 The abbreviations used are:

CRD: carbohydrate-recognition domain
CFDA-SE: carboxyfluorescein diacetate succinimidyl ester
LPS: lipopolysaccharide
DC-SIGN: dendritic cell-specific ICAM-3–grabbing nonintegrin.

## References

[1] Drickamer K., and Taylor M. E. (2015) Recent insights into structures and functions of C-type lectins in the immune system. Curr. Opin. Struct. Biol. 34, 26–34 10.1016/j.sbi.2015.06.003 26163333 PMC4681411

[2] Taylor M. E., and Drickamer K. (2019) Mammalian sugar-binding receptors: known functions and unexplored roles. FEBS J. 286, 1800–1814 10.1111/febs.14759 30657247 PMC6563452

[3] Howard M., Farrar C. A., and Sacks S. H. (2018) Structural and functional diversity of collectins and ficolins and their relationship to disease. Semin. Immunopathol. 40, 75–85 10.1007/s00281-017-0642-0 28894916 PMC5794833

[4] Gibson A. J., Coffey T. J., and Werling D. (2013) Of creatures great and small: the advantages of farm animal models in immunology research. Front. Immunol. 4, 124 10.3389/fimmu.2013.00124 23750160 PMC3664326

[5] Guzman E., and Montoya M. (2018) Contributions of farm animals to immunology. Front. Vet. Sci. 5, 307 10.3389/fvets.2018.00307 30574508 PMC6292178

[6] Blixt O., Head S., Mondala T., Scanlan C., Huflejt M. E., Alvarez R., Bryan M. C., Fazio F., Calarese D., Stevens J., Razi N., Stevens D. J., Skehel J. J., van Die I., Burton D. R., et al (2004) Printed covalent glycan array for ligand profiling of diverse glycan binding proteins. Proc. Natl. Acad. Sci. U.S.A. 101, 17033–17038 10.1073/pnas.0407902101 15563589 PMC534418

[7] Li Z., and Feizi T. (2018) The neoglycolipid (NGL) technology-based microarrays and future prospects. FEBS Lett. 592, 3976–3991 10.1002/1873-3468.13217 30074246

[8] Ng S., Lin E., Kitov P. I., Tjhung K. F., Gerlits O. O., Deng L., Kasper B., Sood A., Paschal B. M., Zhang P., Ling C. C., Klassen J. S., Noren C. J., Mahal L. K., Woods R. J., et al (2015) Genetically encoded fragment-based discovery of glycopeptide ligands for carbohydrate-binding proteins. J. Am. Chem. Soc. 137, 5248–5251 10.1021/ja511237n 25860443 PMC5553193

[9] Stowell S. R., Arthur C. M., McBride R., Berger O., Razi N., Heimburg-Molinaro J., Rodrigues L. C., Gourdine J. P., Noll A. J., von Gunten S., Smith D. F., Knirel Y. A., Paulson J. C., and Cummings R. D. (2014) Microbial glycan microarrays define key features of host-microbial interactions. Nat. Chem. Biol. 10, 470–476 10.1038/nchembio.1525 24814672 PMC4158828

[10] Zheng R. B., Jégouzo S. A. F., Joe M., Bai Y., Tran H. A., Shen K., Saupe J., Xia L., Ahmed M. F., Liu Y. H., Patil P. S., Tripathi A., Hung S. C., Taylor M. E., Lowary T. L., and Drickamer K. (2017) Insights into interactions of mycobacteria with the host innate immune system from a novel array of synthetic mycobacterial glycans. ACS Chem. Biol. 12, 2990–3002 10.1021/acschembio.7b00797 29048873 PMC5735379

[11] Geissner A., Reinhardt A., Rademacher C., Johannssen T., Monteiro J., Lepenies B., Thépaut M., Fieschi F., Mrázková J., Wimmerova M., Schuhmacher F., Götze S., Grünstein D., Guo X., Hahm H. S., et al (2019) Microbe-focused glycan array screening platform. Proc. Natl. Acad. Sci. U.S.A. 116, 1958–1967 10.1073/pnas.1800853116 30670663 PMC6369816

[12] Pilobello K. T., Krishnamoorthy L., Slawek D., and Mahal L. K. (2005) Development of a lectin microarray for the rapid analysis of protein glycopatterns. Chembiochem. 6, 985–989 10.1002/cbic.200400403 15798991

[13] Hirabayashi J., Kuno A., and Tateno H. (2015) Development and applications of the lectin microarray. Top. Curr. Chem. 367, 105–124 10.1007/128_2014_612 25821171

[14] Weis W. I., Taylor M. E., and Drickamer K. (1998) The C-type lectin superfamily in the immune system. Immunol. Rev. 163, 19–34 10.1111/j.1600-065X.1998.tb01185.x 9700499

[15] Feinberg H., Jégouzo S. A., Rowntree T. J., Guan Y., Brash M. A., Taylor M. E., Weis W. I., and Drickamer K. (2013) Mechanism for recognition of an unusual mycobacterial glycolipid by the macrophage receptor mincle. J. Biol. Chem. 288, 28457–28465 10.1074/jbc.M113.497149 23960080 PMC3789947

[16] Willcocks S., Yamakawa Y., Stalker A., Coffey T. J., Goldammer T., and Werling D. (2006) Identification and gene expression of the bovine C-type lectin Dectin-1. Vet. Immunol. Immunopathol. 113, 234–242 10.1016/j.vetimm.2006.04.007 16797084

[17] Gjerstorff M., Hansen S., Jensen B., Dueholm B., Horn P., Bendixen C., and Holmskov U. (2004) The genes encoding bovine SP-A, SP-D, MBL-A, conglutinin, CL-43 and CL-46 form a distinct collectin locus on *Bos taurus* chromosome 28 (BTA28) at position q.1.8–1.9. Anim. Genet. 35, 333–337 10.1111/j.1365-2052.2004.01167.x 15265076

[18] Yamakawa Y., Pennelegion C., Willcocks S., Stalker A., Machugh N., Burt D., Coffey T. J., and Werling D. (2008) Identification and functional characterization of a bovine orthologue to DC-SIGN. J. Leukoc. Biol. 83, 1396–1403 10.1189/jlb.0807523 18319290

[19] Plato A., Willment J. A., and Brown G. D. (2013) C-type lectin-like receptors of the dectin-1 cluster: ligands and signaling pathways. Int. Rev. Immunol. 32, 134–156 10.3109/08830185.2013.777065 23570314 PMC3634610

[20] Pant S. D., Verschoor C. P., Schenkel F. S., You Q., Kelton D. F., and Karrow N. A. (2014) Bovine CLEC7A genetic variants and their association with seropositivity in Johne's disease ELISA. Gene 537, 302–307 10.1016/j.gene.2013.12.020 24393710

[21] Schatz P. J. (1993) Use of peptide libraries to map the substrate specificity of a peptide-modifying enzyme: a 13-residue consensus peptide specifies biotinylation in *Escherichia coli*. Biotechnology 11, 1138–1143 10.1038/nbt1093-1138 7764094

[22] Smith P. A., Tripp B. C., DiBlasio-Smith E. A., Lu Z., LaVallie E. R., and McCoy J. M. (1998) A plasmid expression system for quantitative in vivo biotinylation of thioredoxin fusion proteins in *Escherichia coli*. Nucleic Acids Res. 26, 1414–1420 10.1093/nar/26.6.1414 9490786 PMC147411

[23] Taylor M. E., Bezouska K., and Drickamer K. (1992) Contribution to ligand binding by multiple carbohydrate-recognition domains in the macrophage mannose receptor. J. Biol. Chem. 267, 1719–1726 1730714

[24] Fadden A. J., Holt O. J., and Drickamer K. (2003) Molecular characterisation of the rat Kupffer cell glycoprotein receptor. Glycobiology 13, 529–537 10.1093/glycob/cwg068 12672702

[25] Jang S., Ohtani K., Fukuoh A., Yoshizaki T., Fukuda M., Motomura W., Mori K., Fukuzawa J., Kitamoto N., Yoshida I., Suzuki Y., and Wakamiya N. (2009) Scavenger receptor collectin placenta 1 (CL-P1) predominantly mediates zymosan phagocytosis by human vascular endothelial cells. J. Biol. Chem. 284, 3956–3965 10.1074/jbc.M807477200 19073604

[26] Fuller M. E., Streger S. H., Rothmel R. K., Mailloux B. J., Hall J. A., Onstott T. C., Fredrickson J. K., Balkwill D. L., and DeFlaun M. F. (2000) Development of a vital fluorescent staining method for monitoring bacterial transport in subsurface environments. Appl. Environ. Microbiol. 66, 4486–4496 10.1128/AEM.66.10.4486-4496.2000 11010903 PMC92329

[27] Valdivia R. H., Cormack B. P., and Falkow S. (2006) The uses of green fluorescent protein in prokaryotes. Methods Biochem. Anal. 47, 163–178 16335713

[28] Klein G., Lindner B., Brade H., and Raina S. (2011) Molecular basis of lipopolysaccharide heterogeneity in *Escherichia coli*: envelope stress-responsive regulators control the incorporation of glycoforms with a third 3-deoxy-α-d-manno-oct-2-ulosonic acid and rhamnose. J. Biol. Chem. 286, 42787–42807 10.1074/jbc.M111.291799 22021036 PMC3234843

[29] Heinrichs D. E., Yethon J. A., and Whitfield C. (1998) Molecular basis for structural diversity in the core regions of the lipopolysaccharides of *Escherichia coli* and *Salmonella enterica*. Mol. Microbiol. 30, 221–232 10.1046/j.1365-2958.1998.01063.x 9791168

[30] Widmalm G., and Leontein K. (1993) Structural studies of the *Escherichia coli* O127 O-antigen polysaccharide. Carbohydr. Res. 247, 255–262 10.1016/0008-6215(93)84258-8 7693348

[31] Nishiuchi Y., Doe M., Hotta H., and Kobayashi K. (2000) Structure and serologic properties of *O*-specific polysaccharide from *Citrobacter freundii* possessing cross-reactivity with *Escherichia coli* O157:H7. FEMS Immunol. Med. Microbiol. 28, 163–171 10.1111/j.1574-695X.2000.tb01472.x 10799808

[32] Whitfield C., Szymanski C. M., and Aebi M. (2015) in Essentials of Glycobiology (Varki A., Cummings R. D., Esko J. D., Stanley P., Hart G. W., Aebi M., Darvill A. G., Kinoshita T., Packer N. H., Prestegard J. H., Schnaar R. L., and Seeberger P. H., eds) pp. 265–282, Cold Spring Harbor Laboratory Press, Cold Spring Harbor, NY 27010055

[33] Kuhn H. M., Meier-Dieter U., and Mayer H. (1988) ECA, the enterobacterial common antigen. FEMS Microbiol. Rev. 4, 195–222 10.1111/j.1574-6968.1988.tb02743.x 3078744

[34] Clements A., Gaboriaud F., Duval J. F., Farn J. L., Jenney A. W., Lithgow T., Wijburg O. L., Hartland E. L., and Strugnell R. A. (2008) The major surface-associated saccharides of *Klebsiella pneumoniae* contribute to host cell association. PLoS ONE 3, e3817 10.1371/journal.pone.0003817 19043570 PMC2583945

[35] Vinogradov E., Frirdich E., MacLean L. L., Perry M. B., Petersen B. O., Duus J. Ø., and Whitfield C. (2002) Structures of lipopolysaccharides from *Klebsiella pneumoniae*: elucidation of the structure of the linkage region between core and polysaccharide O chain and identification of the residues at the nonreducing termini of the O chains. J. Biol. Chem. 277, 25070–25081 10.1074/jbc.M202683200 11986326

[36] Feinberg H., Taylor M. E., Razi N., McBride R., Knirel Y. A., Graham S. A., Drickamer K., and Weis W. I. (2011) Structural basis for langerin recognition of diverse pathogen and mammalian glycans through a single binding site. J. Mol. Biol. 405, 1027–1039 10.1016/j.jmb.2010.11.039 21112338 PMC3065333

[37] Mistretta N., Brossaud M., Telles F., Sanchez V., Talaga P., and Rokbi B. (2019) Glycosylation of *Staphylococcus aureus* cell wall teichoic acid is influenced by environmental conditions. Sci. Rep. 9, 3212 10.1038/s41598-019-39929-1 30824758 PMC6397182

[38] East L., Rushton S., Taylor M. E., and Isacke C. M. (2002) Characterization of sugar binding by the mannose receptor family member, Endo-180. J. Biol. Chem. 277, 50469–50475 10.1074/jbc.M208985200 12399458

[39] Brown S., Santa Maria J. P. Jr, and Walker S. (2013) Wall teichoic acids of Gram-positive bacteria. Annu. Rev. Microbiol. 67, 313–336 10.1146/annurev-micro-092412-155620 24024634 PMC3883102

[40] Stambach N. S., and Taylor M. E. (2003) Characterization of carbohydrate recognition by langerin, a C-type lectin of Langerhans cell. Glycobiology 13, 401–410 10.1093/glycob/cwg045 12626394

[41] Powlesland A. S., Fisch T., Taylor M. E., Smith D. F., Tissot B., Dell A., Pöhlmann S., and Drickamer K. (2008) A novel mechanism for LSECtin binding to Ebola virus surface glycoprotein through truncated glycans. J. Biol. Chem. 283, 593–602 10.1074/jbc.M706292200 17984090 PMC2275798

[42] Ng K. K., Drickamer K., and Weis W. I. (1996) Structural analysis of monosaccharide recognition by rat liver mannose-binding protein. J. Biol. Chem. 271, 663–674 10.1074/jbc.271.2.663 8557671

[43] Hansen S., Holm D., Moeller V., Vitved L., Bendixen C., Reid K. B., Skjoedt K., and Holmskov U. (2002) CL-46, a novel collectin highly expressed in bovine thymus and liver. J. Immunol. 169, 5726–5734 10.4049/jimmunol.169.10.5726 12421952

[44] Chatterjee D., and Khoo K. (2001) The surface glycopeptidolipids of mycobacteria: structures and biological of properties. Cell. Mol. Life Sci. 58, 2018–2042 10.1007/PL00000834 11814054 PMC11337330

[45] Feinberg H., Jégouzo S. A. F., Rex M. J., Drickamer K., Weis W. I., and Taylor M. E. (2017) Mechanism of pathogen recognition by human dectin-2. J. Biol. Chem. 292, 13402–13414 10.1074/jbc.M117.799080 28652405 PMC5555199

[46] Yonekawa A., Saijo S., Hoshino Y., Miyake Y., Ishikawa E., Suzukawa M., Inoue H., Tanaka M., Yoneyama M., Oh-Hora M., Akashi K., and Yamasaki S. (2014) Dectin-2 is a direct receptor for mannose-capped lipoarabinomannan of mycobacteria. Immunity 41, 402–413 10.1016/j.immuni.2014.08.005 25176311

[47] Killick K. E., Ní Cheallaigh C., O'Farrelly C., Hokamp K., MacHugh D. E., and Harris J. (2013) Receptor-mediated recognition of mycobacterial pathogens. Cell Microbiol. 15, 1484–1495 10.1111/cmi.12161 23795683

[48] Lang R. (2013) Recognition of the mycobacterial cord factor by mincle: relevance for granuloma formation and resistance to tuberculosis. Front. Immunol. 4, 5 10.3389/fimmu.2013.00005 23355839 PMC3553576

[49] Fulton K. M., Li J., Tomas J. M., Smith J. C., and Twine S. M. (2018) Characterizing bacterial glycoproteins with LC-MS. Expert Rev. Proteomics 15, 203–216 10.1080/14789450.2018.1435276 29400572

[50] Coombs P. J., Graham S. A., Drickamer K., and Taylor M. E. (2005) Selective binding of the scavenger receptor C-type lectin to Lewis^x^ trisaccharide and related glycan ligands. J. Biol. Chem. 280, 22993–22999 10.1074/jbc.M504197200 15845541

[51] Ram S., Lewis L. A., and Rice P. A. (2010) Infections of people with complement deficiencies and patients who have undergone splenectomy. Clin. Microbiol. Rev. 23, 740–780 10.1128/CMR.00048-09 20930072 PMC2952982

[52] Arthur C. M., Rodrigues L. C., Baruffi M. D., Sullivan H. C., Heimburg-Molinaro J., Smith D. F., Cummings R. D., and Stowell S. R. (2015) Examining galectin binding specificity using glycan microarrays. Methods Mol. Biol. 1207, 115–131 10.1007/978-1-4939-1396-1_8 25253137 PMC5755360

[53] Pröpster J. M., Yang F., Ernst B., Allain F. H., and Schubert M. (2015) Functional Siglec lectin domains from soluble expression in the cytoplasm of *Escherichia coli*. Protein Expr. Purif. 109, 14–22 10.1016/j.pep.2015.01.005 25623398

[54] Madeira F., Park Y. M., Lee J., Buso N., Gur T., Madhusoodanan N., Basutkar P., Tivey A. R. N., Potter S. C., Finn R. D., and Lopez R. (2019) The EMBL-EBI search and sequence analysis tools APIs in 2019. Nucleic Acids Res. 47, W636–W641 10.1093/nar/gkz268 30976793 PMC6602479

[55] Huson D. H., and Scornavacca C. (2012) Dendroscope 3: an interactive tool for rooted phylogenetic trees and networks. Syst. Biol. 61, 1061–1067 10.1093/sysbio/sys062 22780991

[56] Eisenberg S. P., Evans R. J., Arend W. P., Verderber E., Brewer M. T., Hannum C. H., and Thompson R. C. (1990) Primary structure and functional expression from complementary DNA of a human interleukin-1 receptor antagonist. Nature 343, 341–346 10.1038/343341a0 2137201

[57] Fornstedt N., and Porath J. (1975) Characterization studies on a new lectin found in seeds of *Vicia ervilia*. FEBS Lett. 57, 187–191 10.1016/0014-5793(75)80713-7 1175787

[58] Young J. C., Clements A., Lang A. E., Garnett J. A., Munera D., Arbeloa A., Pearson J., Hartland E. L., Matthews S. J., Mousnier A., Barry D. J., Way M., Schlosser A., Aktories K., and Frankel G. (2014) The *Escherichia coli* effector EspJ blocks Src kinase activity via amidation and ADP ribosylation. Nat. Commun. 5, 5887 10.1038/ncomms6887 25523213 PMC4284639

[59] Mavridou D. A., Gonzalez D., Clements A., and Foster K. R. (2016) The pUltra plasmid series: a robust and flexible tool for fluorescent labeling of Enterobacteria. Plasmid 87–88, 65–71 10.1016/j.plasmid.2016.09.005 27693407

[60] Carroll P., Schreuder L. J., Muwanguzi-Karugaba J., Wiles S., Robertson B. D., Ripoll J., Ward T. H., Bancroft G. J., Schaible U. E., and Parish T. (2010) Sensitive detection of gene expression in mycobacteria under replicating and nonreplicating conditions using optimized far-red reporters. PLoS ONE 5, e9823 10.1371/journal.pone.0009823 20352111 PMC2843721

