# Supplementary material for: Mammalian lectin arrays for screening host–microbe interactions
Source: J Biol Chem. 2020 Feb 24;295(14):4541–55. doi: 10.1074/jbc.RA120.012783 (PMC7135977; doi:10.1074/jbc.RA120.012783)
Supplement: Supporting Information [file supp_295_14_4541__index.html]

Mammalian lectin arrays for screening host-microbe interactions — Mammalian lectin array — Mammalian lectin arrays for screening host–microbe interactions — Mammalian lectin array — Supporting Information 

# Mammalian lectin arrays for screening host–microbe interactions

## Supporting Information

- Supporting Information (to be published online) - Figures S1-S28 Table S1
