# Supplementary material for: Mammalian lectin arrays for screening host–microbe interactions
Source: J Biol Chem. 2020 Feb 24;295(14):4541–55. doi: 10.1074/jbc.RA120.012783 (PMC7135977; doi:10.1074/jbc.RA120.012783)
Supplement: Supporting Information [file supp_RA120.012783_158421_1_supp_475667_q5p36j.pdf]

Supporting Information

Mammalian lectin arrays for screening host-microbe interactions

Sabine A.F. Jégouzo, Conor Nelson, Thomas Hardwick, S. T. Angel Wong, Noel Kuan Kiat Lau, Gaik Kin Emily Neoh, Rocío Castellanos-Rueda, Zhiyao Huang, Benjamin Mignot, Aanya Hirdaramani, Annie Howitt, Kathryn Frewin, Zheng Shen (沈 诤), Rhys J. Fox, Rachel Wong, Momoko Ando, Lauren Emony, Henderson Zhu, Angela Holder, Dirk Werling, Nitya Krishnan, Brian D. Robertson, Abigail Clements, Maureen E. Taylor, and Kurt Drickamer

Figure S1. Sequence aligment of cow C-type CRDs used for lectin array.

Figures S2-S28. cDNA sequences for individual cow CRDs with PCR primers used for amplification.

Table S1. Bacterial strains used in this study.

Figure S1. Aligned sequences of bovine C-type CRDs.

Sequence alignments were adjusted manually to highlight the pattern of conserved amino acid residues highlighted in *yellow*. Additional shading indicates the five amino acid residues that form the conserved Ca^{2+}-binding site which in turn forms the primary monosaccharide-binding site, in *magenta*, and the four amino acid residues that form a secondary Ca^{2+}-binding site, in *cyan*.

```
MBP-A                             SGKKLYVTNREKMPFSSVKALCTALGATVATPKNAEENKAIQDMAS------DTAFLGI
MBP-C                             VGKKAFFTNGKKMPFNEVKTLCAQFQGRVATPMNAEENRALKDLVT------EEAFLGI
CL43                              VGEKIFKTAGAVKSYSDAEQLCREAKGQLASPRSSAENEAVTQLVR---AKNKHAYLSM
SP-D                              VGEKIFKTVGSEKTFQDAQQICTQAGGQLPSPRSGAENEALTQLAT---AQNKAAFLSM
CL46                              VGKKIFKTAGAVKSYSDAQQLCREAKGQLASPRSAAENEAVAQLVR---AKNNDAFLSM
Conglutinin                       VGEKIFKTAGAVKSYSDAEQLCREAKGQLASPRSSAENEAVTQMVR---AQEKNAYLSM
COL-K1                            TEQKMYLLVKEEKRYLDAQLACQGRGGTLSMPKDEAANALLAAYIT--QAGLARVFIGI
SP-A                              VGEKVFSTNGQSVNFDAIKELCARVGGHIAAPRSPEENEAIVSIVK---KYNTYAYLGL
COL-L1                            TEEKFYYIVQEEKNYRESLTHCRIRGGMLAMPKDEAANTLLADYVS--KSGFFRVFIGV
MMR CRD4            KCPEDWGASSKTSLCFKLFAKGKHEKKTWFESRDFCRALGGDLASINSKEEQQAIWRLVTASGSYHELFWLGL
MMR CRD5            PHDNLPVTDDGWVIYKDYQYYFSKEKATMDKAREFCKRNFGDLVSIRSESEKKFLWVNRN---DVQPAYFIGL
Langerin                 QVVSQGWKYFGGHFYYFSKISKTWYSAQQICISRDSHLTSVTSEREQEFLYRTAG-----GLPYWIGL
DC-SIGN               GLCHPCPQNWEFFDGSCYFFSWTQSDWRSAVSACLLIGAHLVIIESTEEEKFLNFWYP---RNNKPTWIGL
Prolectin           LDCTRVTCPEGWLPFQGKCYYFSPSTKSWDEARKFCQENYSHLVIISNSDEQDFVAKAHG----SPRVYWLGL
CD23                NGSVCNTCPEAWIYFQKKCYYFGEGAKKWIQARYACENLHGRLVSIHSPEEQDFLTKRAN-----WRGSWIGL
LSECtin               SSCKECPESWLPFQGSCYFFSTLRATWVEAQQHCERSGAHLVIVGGLEEQGFLSRNTR-----GRGYWLGL
Endo180 CRD2             VECEPSWQPFQGHCYRLQAEKRSWQESKKMCLRGGGDLLSIHSMAELEFITKQIK---QEVEELWIGL
Mincle   LSCYNDGSGSVK----NCCPLKWFHFQSSCYLFSPDTMSWRASLKNCSSMGAHLVVINTQEEQEFLYYTKP----RKKEFYIGL
Dectin-2 LTCFSEGTRVTE-KIWGCCPGTWKPFGSSCYFISSEENFWAKSEQNCIGMGAHLVVINTETEQDFIIQQLN----KTFSYFLGL
Dectin-1  SVIPTKALTTTGVFSSSCPPNWITHEDSCYLFSTLLDSWDGSKRQCFQLGSHLLKIDSSKELEFISRQVS--SQPDHSFWIGL
ASGPR1    SCQMAVLQGNG-SEKACCPVNWIDYEGSCYWFSRSGKPWPEAEKYCQLENAHLVVVGSWEEQKFI-QHHM----GPVNTWIGL
ASGPR2    TCQMAHFQSNG---TECCPVNWVDHDGSCYWFSRSGKPWLEAEKYCQLENAHLVVINSREEQKFI-VQHT----NPFRVWIGL
MGL      LTCKMDALKSNGSQNTACCPANWLEHEGHCYWFSSLRKPWPEAEKDCQLKNAQLVVINSRDEQDFI-QANL----HPYFTWMGL
SRCL                   EDNGCLPYWKNFTDKCYYFSTERDFFEDAKLFCERMSSHLVFINTGEEQQWIKNQMV----AKQNYWIGL
KCR                               YSGSLYYFSSAKKTWQEAEQFCVSHGAHLASVTSEEEKTFLIQFTS-----SVYHWIGL


MBP-A         TDEVTEGQKKFMYVT----GGRLGYSNWKKNEPNNYGSG-----EDCVSLL----PDGLWNDISCSSS-FLAICEFP
MBP-C         TDIETEG--KFVDLT----GKGVTYQNWNDGEPNNASPG-----EHCVTLL----SDGTWNDIACSAS-FLTVCEFSL
CL43          NDISKEG--KFTYPT----GGSLDYSNWAPGEPNNRAKD--EGPENCLEIY----SDGNWNDIECREE-RLVICEF
SP-D          SDTRKEG--TFIYPT----GEPLVYSNWAPQEPNNDGGS-----ENCVEIF----PNGKWNDKVCGEQ-RLVICEF
CL46          NDISTEG--KFTYPT----GESLVYSNWASGEPNNNNAG---QPENCVQIY----REGKWNDVPCSEP-LLVICEF
Conlgutinin   NDQSTEG--RFTYPT----GEILVYSNWADGEPNNSDEG---QPENCVEIF----PDGKWNDVPCSKQ-LLVICEF
COL-K1        NDLEREG--AFVYAD---RSPMQTFSKWRSGEPNNAYD-----EEDCVELV----ASGGWNDVACHLT-MHFLCEFDKEHV
SP-A          VEGPTAG--DFYYLD----GAPVNYTNWYPGEPRGRGK------EKCVEIY----TDGQWNDKNCLQY-RLAICEF
COL-L1        NDLEREG--QYVFTD---NTPLQNYSNWKEGEPSDPYGH-----EDCVEML----SSGRWNDTECHLT-MYFVCEFIKKKK
MMR CRD4      TYGSPSE--GFTWSD----GSPVSYENWAYGEPNNYQNV-----EYCGELK--GDPGMSWNDINCEHL-NNWICQI
MMR CRD5       LISLDK--KFIWMD----GSKVDYVAWATGEPNFANDD-----ENCVTMY---SNSGFWNDINCGYP-NAFICQR
Langerin      TKAGSEG--DWHWVDGTPYNKVQSEKFWIPGEPNNVGNN-----EHCVTLK--TSLLRSWNDASCDNT-FLFICKRSYKPSEP
DC-SIGN       SDHHSEG--SWRWVD---DSPVQL-SFWKKGEPNNHGD------EDCVELH-----NDGWNDGRCVTE-NPWICEKPSVPCPVL
Prolectin     NDRNVEG--DWRWLD----GSPVTLSFWDPQEPNNLYNN-----ENCASMN----KGGTWNDLSCDKT-TYWICERK---CSC
CD23          RDLDIEG--EFIWMD----NQPLDYSNWQPGEPNDAGQG-----ENCVMML----GSGKWNDAFCGSELHGWVCDRL-ATCG
LSECtin    RAVRKVRRIQ--GYQWVD----GVALSFSHWNRGEPNDSMGR-----EDCIMML----RTGMWNDAPCDNENDNWICEKR-LSC
Endo180 CRD2  NDLKLQM--NFEWSD----GSLVSFTHWHPFEPNNFRDSL----EDCVTIW---GPEGRWNDSPCNQS-LPSICKK
Mincle        TDQVTEG--QWQWVD--GTPFTKSLSFWDAEEPNNLVTV-----EDCATIRDSSNPRQNWNDVPCFFN-MFRVCEMPERKIG
Dectin-2      SDPQGNG--NWQWID--QTPYKENVRFWHQNEPNFSA-------EECASVVFWDGRGWGWNDVFCDSK-RKSICEMKKIYL
Dectin-1      SRRQTEE--PWLWED--GSTLLSNLFQIRSTVTEKDSS------HNCAWIH-----VSDIYDQLCSVH-SYSICEKKLSV
ASGPR1        MDQ--NG--PWKWVD--GTDYETGFKNWAPEQPDDWYGHGLGGGEDCAHIT----VDGRWNDDVCLRP-YRWVCEAQRDGGNDS
ASGPR2        TDS--DG--SWKWVD--GTDYKHSYKNWDPAQPDDWRGHELGASEDCAEIR----WDGRWNDDFCQQV-KRWVCETKRNITM
MGL           SDP--DG--VWKWVD--GSDYETNIKNWKPGQPDDFHGHGLGGGEDCAHFY----PDGEWNDDACQRL-YYWICEAGLSQVHNKMH
SRCL          TDLEQEN--EWRWLD--GTLLEYK--NWKAGQPDNW-GHGHGPGEDCAGLI----NFGQWNDFPCEDM-NHFICEKDRERELAITL
KCR           TDHGTEG--HWRWTDGTAFDRARSRAFWAENQPDNWQHG-IGQSEDCVQMQ------QKWNDISCSTL-CRWICKKPMVQL
```

PCR primers and sequences of cow cDNA of current lectin array proteins

Sequences of cloned CRDs are shown in black. Appended initiation sequences and biotin tags are indicated in *red*. Sequences of primers used for PCR amplification are shown in *green*. Variations in the sequences compared to the genomic sequence in the National Center for Biotechnology Information database are highlighted in *blue*.

Figure S2. Mannose-binding protein A CRD (MBP-A)

```
                                          MetAlaSerGlyLysLysLeuTyrValThrAsnAr
ggatccgatcttggaggatgattaaatggcctctgggaagaagctgtatgtgaccaatcg
ggatccgatcttggaggatgattaaatggcctctgggaagaagctgtatgtgacc

gGluLysMetProPheSerSerValLysAlaLeuCysThrAlaLeuGlyAlaThrValAl
tgaaaagatgcctttttccagcgtgaaggctctgtgcactgcacttggggctaccgtggc

aThrProLysAsnAlaGluGluAsnLysAlaIleGlnAspMetAlaSerAspThrAlaPh
cacacccaagaatgcagaggagaacaaagccatccaggacatggcctctgataccgcctt

eLeuGlyIleThrAspGluValThrGluGlyGlnPheMetTyrValThrGlyGlyArgLe
cctgggcatcacagatgaggtgactgaagggcagtttatgtatgtaactggaggaaggct

uGlyTyrSerAsnTrpLysLysAsnGluProAsnAsnTyrGlySerGlyGluAspCysVa
aggctacagcaactggaagaagaatgaacccaataactatggctcaggggaggactgtgt

lSerLeuLeuProAspGlyLeuTrpAsnAspIleSerCysSerSerSerPheLeuAlaIl
gagcctcttaccagacgggctctggaatgacatctcctgttcttcctccttcttggccat
                                                    aaccggta

eCysGluPheProAlaLeuAsnAspIlePheGluAlaGlnLysIleGluTrpHisGlu**
ctgtgaatttccagccctgaatgacatcttcgaagcacagaaaatcgagtggcatgagta
gacacttaaaggtcgggacttactgtagaagcttcgtgtcttttagctcaccgtactcat

*
ggaattc
ccttaag
```

Figure S3. Mannose-binding protein C CRD (MBP-C)

```
                                          MetAlaValGlyLysLysAlaPhePheThrAsnGl
ggatccgatcttggaggatgattaaatggccgttgggaagaaggcatttttttaccaatgg
ggatccgatcttggaggatgattaaatggccgttgggaagaaggcattttttacc

yLysLysMetProPheAsnGluValLysThrLeuCysAlaGlnPheGlnGlyArgValAl
taaaaagatgccttttaatgaagtgaagactctgtgtgcacagttccagggccgtgtggc

aThrProMetAsnAlaGluGluAsnArgAlaLeuLysAspLeuValThrGluGluAlaPh
cacccctatgaatgctgaagaaaacagggccctcaaggatttagtcactgaagaggcctt

eLeuGlyIleThrAspGlnGluThrGluGlyLysPheValAspLeuThrGlyLysGlyVa
cctgggcatcacagatcaggagactgaaggcaaatttgtggatctgacaggaaagggggt

lThrTyrGlnAsnTrpAsnAspGlyGluProAsnAsnAlaSerProGlyGluHisCysVa
gacctaccaaaactggaatgatggcgagcctaacaacgcttctcctggggagcactgtgt

lThrLeuLeuSerAspGlyThrTrpAsnAspIleAlaCysSerAlaSerPheLeuThrVa
gacacttctgtcggacggcacatggaatgacatcgcttgttccgcctcctttttgaccgt
                                                    aactggca

lCysGluPheSerLeuLeuAsnAspIlePheGluAlaGlnLysIleGluTrpHisGlu**
ctgtgaattctctctcctgaatgacatcttcgaagcacagaaaatcgagtggcatgagta
gacacttaagagagaggacttactgtagaagcttcgtgtcttttagctcaccgtactcat

*
gaagctt
cttcgaa
```

Figure S4. Collectin 43 CRD (CL-43)

```
                         MetAlaValGlyGluLysIlePheLysThrAlaGl
ggatccgatcttggaggatgattaaatggccgtcggggagaagatcttcaagacggcagg
ggatccgatcttggaggatgattaaatggccgtcggggagaagatcttcaagacg

yAlaValLysSerTyrSerAspAlaGluGlnLeuCysArgGluAlaLysGlyGlnLeuAl
tgctgtaaagtcatattcggatgcagagcagctctgcagagaggctaagggacagctggc

aSerProArgSerSerAlaGluAsnGluAlaValThrGlnLeuValArgAlaLysAsnLy
ctccccacgctcttcagccgagaacgaggccgtgacacagctggtcagagccaagaacaa

sHisAlaTyrLeuSerMetAsnAspIleSerLysGluGlyLysPheThrTyrProThrGl
gcatgcttacctgagcatgaatgacatctccaaagagggcaagttcacctacccaacggg

yGlySerLeuAspTyrSerAsnTrpAlaProGlyGluProAsnAsnArgAlaLysAspGl
ggggtcactggactattccaactgggcccccggggagcccaacaatagggcaaaagacga

uGlyProGluAsnCysLeuGluIleTyrSerAspGlyAsnTrpAsnAspIleGluCysAr
aggcccagagaactgtcttgagatctattccgatggcaattggaatgacatagaatgcag

gGluGluArgLeuValIleCysGluPheLeuAsnAspIlePheGluAlaGlnLysIleGl
ggaggagcgcctcgtgatctgtgagttcctgaatgacatcttcgaagcacagaaaatcga
    ctcgcggagcactagacactcaaggacttactgtagaagcttcgtgtcttttagct

uTrpHisGlu***
gtggcatgagtag
caccgtactcatctt
```

Figure S5. Surfactant Protein D CRD (SP-D)

```
                                                 ggatccgatct
                                                 ggatccgatct

              MetAlaValGlyGluLysIlePheLysThrGluGlySerGluLysT
tggaggatgattaaatggccgtcggggagaagatctttaagacggaaggctctgaaaaaa
tggaggatgattaaatggccgtcggggagaagatctttaagacg

hrPheGlnAspAlaGlnGlnIleCysThrGlnAlaGlyGlyGlnLeuProSerProArgS
cgtttcaggatgcccagcagatctgcacacaggctggaggacagttgccctccccacgtt

erAlaAlaGluAsnGluAlaLeuThrGlnLeuAlaThrAlaGlnAsnLysAlaAlaPheL
ctgcagctgaaaacgaggccttgactcagctggccacagcccagaacaaggctgctttcc

euSerMetSerAspThrArgLysGluGlyThrPheIleTyrProThrGlyGluProLeuV
tgagcatgagcgacaccaggaaggagggtactttcatctaccccacgggggagcccctgg

alTyrSerAsnTrpAlaProGlnGluProAsnAsnAspGlyGlySerGluAsnCysValG
tctattccaactgggcccccaggagcccaacaatgatggcggctcagagaactgtgtgg

luIlePheProAsnGlyLysTrpAsnAspLysValCysGlyGluGlnArgLeuValIleC
agatctttcccaacggcaagtggaatgacaaggtctgcggagagcagcgcctcgtgatct
                                            gtcgcggagcactaga

ysGluPheLeuAsnAspIlePheGluAlaGlnLysIleGluTrpHisGlu***
gcgagttcctgaatgacatcttcgaagcacagaaaatcgagtggcatgagtaggaattc
cgctcaaggacttactgtagaagcttcgtgtcttttagctcaccgtactcatccttaag
```

Figure S6. Collectin 46 CRD (CL-46)

```
                         MetAlaValGlyLysLysIlePheLysThrAlaGl
ggatccgatcttggaggatgattaaatggccgttgggaagaagatcttcaagacggcagg
ggatccgatcttggaggatgattaaatggccgttgggaagaagatcttcaagacg

yAlaValLysSerTyrSerAspAlaGlnGlnIleCysArgGluAlaLysGlyGlnLeuAl
tgctgtaaaatcatattcggatgcccagcagatctgcagagaggctaagggacagctggc

aSerProArgSerAlaAlaGluAsnGluAlaValAlaGlnLeuValArgAlaLysAsnAs
ctccccacgctctgcagctgagaacgaggccgtggcacagctggtcagagccaagaacaa

nAspAlaPheLeuSerMetAsnAspIleSerThrGluGlyLysPheThrTyrProThrGl
tgatgctttcctgagcatgaatgacatctccacggagggcaagttcacctaccccacggg

yGluSerLeuValTyrSerAsnTrpAlaSerGlyGluProAsnAsnAsnAsnAlaGlyGl
ggagtcactggtctattccaactgggccagtggggagcccaacaacaacaatgctggaca

nProGluAsnCysValGlnIleTyrArgGluGlyLysTrpAsnAspValProCysSerGl
accagagaactgtgtgcagatctatcgggagggcaagtggaatgacgtaccctgcagtga

uProLeuLeuValIleCysGluPheLeuAsnAspIlePheGluAlaGlnLysIleGluTr
gccactccttgtgatctgcgagtttctgaatgacatcttcgaagcacagaaaatcgagtg
 ggtgaggaacactagacgctcaaagacttactgtagaagcttcgtgtcttttagctcac

pHisGlu***
gcatgagtagaa
cgtactcatctt
```

Figure S7. Conglutinin CRD

```
                         MetAlaValGlyGluLysIlePheLysThrAlaGl
ggatccgatcttggaggatgattaaatggccgtcggggagaagatcttcaagacagcagg
ggatccgatcttggaggatgattaaatggccgtcggggagaagatcttcaagaca

yAlaValLysSerTyrSerAspAlaGluGlnLeuCysArgGluAlaLysGlyGlnLeuAl
tgctgtaaagtcatattcagatgcagagcagctctgcagagaggctaagggacagctggc

aSerProArgSerSerAlaGluAsnGluAlaValThrGlnMetValArgAlaGlnGluLy
ctccccacgctcttcagccgagaacgaggccgtgacacagatggtcagagcccaggaaaa

sAsnAlaTyrLeuSerMetAsnAspIleSerThrGluGlyArgPheThrTyrProThrGl
gaatgcttacctgagcatgaatgacatctccacggaggggaggttcacttaccccactgg

yGluIleLeuValTyrSerAsnTrpAlaAspGlyGluProAsnAsnSerAspGluGlyGl
ggaaatactggtctattccaactgggccgatggggagcccaacaacagtgatgagggaca

nProGluAsnCysValGluIlePheProAspGlyLysTrpAsnAspValProCysSerLy
accagagaactgtgtggaaatctttcctgatggcaagtggaatgacgtaccctgcagtaa

sGlnLeuLeuValIleCysGluPheLeuAsnAspIlePheGluAlaGlnLysIleGluTr
gcaactccttgtgatctgcgagtttctgaatgacatcttcgaagcacagaaaatcgagtg
  gttgaggaacactagacgctcaaagacttactgtagaagcttcgtgtcttttagctca

pHisGlu***
gcatgagtaggaa
ccgtactcatctt
```

Figure S8. Collectin K1 CRD (ColK1)

```
                                            ggatccgatcttggag
                                            ggatccgatcttggag

      MetAlaThrGluGlnLysMetTyrLeuLeuValLysGluGluLysArgTyr
gatgattaaatggccacggagcagaagatgtacctgctggtgaaggaggagaagcgctac
gatgattaaatggccacggagcagaagatgtacctgctg

LeuAspAlaGlnLeuAlaCysGlnGlyArgGlyGlyThrLeuSerMetProLysAspGlu
ctggacgcgcagctggcctgccagggccggggcggcacactgagcatgcccaaggacgag

AlaAlaAsnAlaLeuLeuAlaAlaTyrIleThrGlnAlaGlyLeuAlaArgValPheIle
gccgccaacgcgctgctggccgcctacatcacgcaggccggcctggcccgcgtcttcatc

GlyIleAsnAspLeuGluArgGluGlyAlaPheValTyrAlaAspArgSerProMetGln
ggcatcaatgacctggagagggaaggcgcgttcgtctacgcggaccgctcgccgatgcag

ThrPheSerLysTrpArgSerGlyGluProAsnAsnAlaTyrAspGluGluAspCysVal
accttcagcaagtggcgcagcggggaacccaacaacgcctacgacgaggaggactgcgtg

GluLeuValAlaSerGlyGlyTrpAsnAspValAlaCysHisLeuThrMetHisPheLeu
gagctggtggcctcagggggttggaacgacgtggcctgccaccttaccatgcacttcctc

CysGluPheAspLysGluHisValLeuAsnAspIlePheGluAlaGlnLysIleGluTrp
tgcgagttcgacaaggagcacgtgctgaatgacatcttcgaagcacagaaaatcgagtgg
acgctcaagctgttcctcgtgcacgacttactgtagaagcttcgtgtcttttagctcacc

HisGlu***
catgagtaggaattc
gtactcatccttaag
```

Figure S9. Surfactant Protein A CRD (SP-A)

```
                                   MetAlaValGlyGluLysValPheSer
     ggatccgatcttggaggatgattaaatggccgtgggagagaaggtcttctct
     ggatccgatcttggaggatgattaaatggccgtgggagagaaggtcttctct

ThrAsnGlyGlnSerValAsnPheAspAlaIleLysGluLeuCysAlaArgValGlyGly
accaatgggcagtcagtcaattttgatgccattaaagagttatgtgccagagtaggtgga
acc

HisIleAlaAlaProArgSerProGluGluAsnGluAlaIleValSerIleValLysLys
catattgctgccccgaggagtccagaggagaatgaagccattgtgagcatcgtgaagaag

TyrAsnThrTyrAlaTyrLeuGlyLeuValGluGlyProThrAlaGlyAspPheTyrTyr
tacaacacttatgcttacctgggcctggtcgaaggccccaccgctggagacttctattac

LeuAspGlyAlaProValAsnTyrThrAsnTrpTyrProGlyGluProArgGlyArgGly
ctggatggagcccctgtgaattataccaattggtacccaggggagcccaggggccggggt

LysGluLysCysValGluIleTyrThrAspGlyGlnTrpAsnAspLysAsnCysLeuGln
aaagagaagtgtgtagaaatatacacagatggtcagtggaatgacaagaactgcctgcag

TyrArgLeuAlaIleCysGluPheLeuAsnAspIlePheGluAlaGlnLysIleGluTrp
taccgactggccatctgtgagttcctgaatgacatcttcgaagcacagaaaatcgagtgg
atggctgaccggtagacactcaaggacttactgtagaagcttcgtgtcttttagctcacc

HisGlu***
catgagtaggaattc
gtactcatccttaag
```

Figure S10. Collectin L1 CRD (ColL1)

```
                                    MetAlaThrGluGluLysPheTyr
            ggatccgatcttggaggatgattaaatggccaccgaagagaaattctac
            ggatccgatcttggaggatgattaaatggccaccgaagagaaattctac

TyrIleValGlnGluGluLysAsnTyrArgGluSerLeuThrHisCysArgIleArgGly
tacatcgtgcaggaggagaagaactacagggaatccctgacccactgccgcatccggggt
tacatcgtgc

GlyMetLeuAlaMetProLysAspGluAlaAlaAsnThrLeuLeuAlaAspTyrValSer
ggaatgctagccatgcccaaggacgaagctgccaacacgctgctggctgactacgtctcc

LysSerGlyPhePheArgValPheIleGlyValAsnAspLeuGluArgGluGlyGlnTyr
aagagtggcttcttccgggtgttcatcggggtgaacgacctggagagggagggtcagtat

ValPheThrAspAsnThrProLeuGlnAsnTyrSerAsnTrpLysGluGlyGluProSer
gtgttcacagataacactcccctgcagaactacagcaactggaaggagggtgagcccagc

AspProTyrGlyHisGluAspCysValGluMetLeuSerSerGlyArgTrpAsnAspThr
gacccctatggccacgaggactgtgtggagatgctgagctcaggcagatggaatgatacc

GluCysHisLeuThrMetTyrPheValCysGluPheIleLysLysLysLysLeuAsnAsp
gagtgccatcttaccatgtactttgtctgtgaattcatcaagaagaaaaagctgaatgac
                        cagacacttaagtagttcttctttttcgacttactg

IlePheGluAlaGlnLysIleGluTrpHisGlu***
atcttcgaagcacagaaaatcgagtggcatgagtagaagctt
tagaagcttcgtgtcttttagctcaccgtactcatcttcgaa
```

Figure S11. Mannose Receptor CRD 4 (MMR CRD 4)

```
                                              MetAlaLysCysPr
                                              atggccaaatgtcc
                                               tggccaaatgtcc

oGluAspTrpGlyAlaSerSerLysThrSerLeuCysPheLysLeuPheAlaLysGlyLy
agaggattggggtgcctccagtaaaacaagcttgtgcttcaaactgtttgcaaaaggaaa
agaggattggggtgcc

sHisGluLysLysThrTrpPheGluSerArgAspPheCysArgAlaLeuGlyGlyAspLe
acatgagaagaaaacgtggtttgaatctcgagattttgtagagctctgggtggagatct

uAlaSerIleAsnSerLysGluGluGlnGlnAlaIleTrpArgLeuValThrAlaSerGl
agctagtatcaatagtaaggaggaacagcaagcaatatggagattagtaacggctagtgg

ySerTyrHisGluLeuPheTrpLeuGlyLeuThrTyrGlySerProSerGluGlyPheTh
aagctaccatgaactattttggttgggactgacatatggaagtccttccgagggctttac

rTrpSerAspGlySerProValSerTyrGluAsnTrpAlaTyrGlyGluProAsnAsnTy
ttggagtgatggatcccctgtgtcatatgaaaattgggcttatggagaacctaataatta

rGlnAsnValGluTyrCysGlyGluLeuLysGlyAspProGlyMetSerTrpAsnAspIl
tcaaaatgttgaatactgtggtgagttaaaaggtgaccctggtatgtcctggaatgacat

eAsnCysGluHisLeuAsnAsnTrpIleCysGlnIleLeuAsnAspIlePheGluAlaGl
taactgtgaacatcttaacaactggatttgccagatactgaatgacatcttcgaagcaca
             gaattgttgacctaaacggtctatgacttactgtagaagcttcgtgt

nLysIleGluTrpHisGlu***
gaaaatcgagtggcatgagtag
cttttagctcaccgtactcatc
```

Figure S12. Mannose Receptor CRDs 4 and 5 (MMR CRD45)

```
                                                 MetAlaLysCysPr
                                                 atggccaaatgtcc
                                                  tggccaaatgtcc

oGluAspTrpGlyAlaSerSerLysThrSerLeuCysPheLysLeuPheAlaLysGlyLy
agaggattggggtgcctccagtaaaacaagcttgtgcttcaaactgtttgcaaaaggaaa
agaggattggggtgcc

sHisGluLysLysThrTrpPheGluSerArgAspPheCysArgAlaLeuGlyGlyAspLe
acatgagaagaaaacgtggtttgaatctcgagattttgtagagctctgggtggagatct

uAlaSerIleAsnSerLysGluGluGlnGlnAlaIleTrpArgLeuValThrAlaSerGl
agctagtatcaatagtaaggaggaacagcaagcaatatggagattagtaacggctagtgg

ySerTyrHisGluLeuPheTrpLeuGlyLeuThrTyrGlySerProSerGluGlyPheTh
aagctaccatgaactattttggttgggactgacatatggaagtccttccgagggctttac

rTrpSerAspGlySerProValSerTyrGluAsnTrpAlaTyrGlyGluProAsnAsnTy
ttggagtgatggatcccctgtgtcatatgaaaattgggcttatggagaacctaataatta

rGlnAsnValGluTyrCysGlyGluLeuLysGlyAspProGlyMetSerTrpAsnAspIl
tcaaaatgttgaatactgtggtgagttaaaaggtgaccctggtatgtcctggaatgacat

eAsnCysGluHisLeuAsnAsnTrpIleCysGlnIleArgLysGlyGlnThrProLysPr
taactgtgaacatcttaacaactggatttgccagatacgaaaaggacaaactcccaaacc

oGluProThrProAlaProHisAspAsnLeuProValThrAspAspGlyTrpValIleTy
tgagccaacaccagctcctcacgacaatctaccagtcaccgatgatgggtgggttattta

rLysAspTyrGlnTyrTyrPheSerLysGluLysAlaThrMetAspLysAlaArgGluPh
caaagactaccagtattatttcagcaaagagaaggcaaccatggacaaggcacgagaatt

eCysLysArgAsnPheGlyAspLeuValSerIleArgSerGluSerGluLysLysPheLe
ttgcaagaggaattttggtgatcttgtttctatccgaagtgaaagtgaaaagaagtttct

uTrpLysTyrValAsnArgAsnAspValGlnProAlaTyrPheIleGlyLeuLeuIleSe
atggaaatatgtgaacaggaatgatgtacagccggcatattttattggtttattgatcag

rLeuAspLysLysPheIleTrpMetAspGlySerLysValAspTyrValAlaTrpAlaTh
cttggataaaaaatttatttggatggatggaagcaaagtggattatgtggcttgggccac

rGlyGluProAsnPheAlaAsnAspAspGluAsnCysValThrMetTyrSerAsnSerGl
aggtgaacccaattttgcaaatgatgatgaaaactgtgtaaccatgtattcaaattcagg

yPheTrpAsnAspIleAsnCysGlyTyrProAsnAlaPheIleCysGlnArgLeuAsnAs
gttttggaatgacattaactgtggttatccaaatgccttcatctgccagcgcctgaatga
                               ggtttacggaagtagacggtcgcggacttact

pIlePheGluAlaGlnLysIleGluTrpHisGlu***
catcttcgaagcacagaaaatcgagtggcatgagtaa
gtagaagcttcgtgtcttttagctcaccgtactcatc
```

Figure S13. Langerin CRD

```
                                            ggatccgatcttggag
                                            ggatccgatcttggag

       MetAlaGlnValValSerGlnGlyTrpLysTyrPheGlyGlyHisPheTyr
gatgattaaatggcccaggtggtttcccaaggctggaagtacttcggggggcacttctat
gatgattaaatggcccaggtggtttcccaaggctggaag

TyrPheSerLysIleSerLysThrTrpTyrSerAlaGlnGlnIleCysIleSerArgAsp
tacttttctaaaatctcgaagacctggtacagtgcccagcagatctgtatatcgagggac

SerHisLeuThrSerValThrSerGluArgGluGlnGluPheLeuTyrArgThrAlaGly
tcccacctgacctcagtgacctcagagcgtgaacaggagttcctctacaggacagcaggc

GlyLeuProTyrTrpIleGlyLeuThrLysAlaGlySerGluGlyAspTrpHisTrpVal
ggacttccctactggatcggcctgaccaaagcagggagcgagggggactggcactgggtg

AspGlyThrProTyrAsnLysValGlnSerGluLysPheTrpIleProGlyGluProAsn
gatggcactccgtacaacaaggtccagagtgagaagttctggattccaggagaacccaac

AsnValGlyAsnAsnGluHisCysValThrLeuLysThrSerLeuLeuArgSerTrpAsn
aacgttgggaacaacgaacactgtgtcaccctaaagacgtccttactgcggtcatggaac

AspAlaPheCysAspAsnThrPheLeuPheIleCysLysArgSerTyrLysProSerGlu
gatgccttctgtgacaatacatttctttttatctgtaagcggtcctataaaccatcagaa
                                       cggtcctataaaccatcagaa

ProLeuAsnAspIlePheGluAlaGlnLysIleGluTrpHisGlu***
ccactgaatgacatcttcgaagcacagaaaatcgagtggcatgagtaggaattc
ccactgaatgacatcttcgaagcacagaaaatcgagtggcatgagtaggaattc
```

Figure S14. DC-SIGN CRD

```
                                            ggatccgatcttggag
                                            ggatccgatcttggag

       MetAlaGlyLeuCysHisProCysProGlnAsnTrpGluPhePheAspGly
gatgattaaatggccggcctgtgccatccttgtcctcagaattgggagtttttcgatgga
gatgattaaatggccggcctgtgccatccttgtcctcag

SerCysTyrPhePheSerTrpThrGlnSerAspTrpArgSerAlaValSerAlaCysLeu
agctgctacttcttctcctggacccagagtgactggagatctgccgtctctgcctgtctg

LeuIleGlyAlaHisLeuValIleIleGluSerThrGluGluGluLysPheLeuAsnPhe
cttattggggcccacctagttatcatcgagagtactgaggaggagaaattcctgaacttt

TrpTyrProArgAsnAsnLysProThrTrpIleGlyLeuSerAspHisHisSerGluGly
tggtatcccagaaataataaacccacctggatcggcctcagtgaccaccacagtgagggt

SerTrpArgTrpValAspAspSerProValGlnLeuSerPheTrpLysLysGlyGluPro
tcctggcggtgggtggatgacagtcctgtccaactcagcttctggaaaaaaggggagccc

AsnAsnHisGlyAspGluAspCysValGluLeuHisAsnAspGlyTrpAsnAspGlyArg
aacaaccacggagatgaggactgtgtggaactgcacaacgatggctggaatgatggcaga

CysValThrGluAsnProTrpIleCysGluLysProSerValProCysProValLeuLeu
tgtgttacagaaaacccctggatctgtgagaagccctcggttccctgcccagtcctcctg
                                 ccctcggttccctgcccagtcctcctg

AsnAspIlePheGluAlaGlnLysIleGluTrpHisGlu***
aatgacatcttcgaagcacagaaaatcgagtggcatgagtaggaattc
aatgacatcttcgaagcacagaaaatcgagtggcatgagtaggaattc
```

Figure S15. Prolectin CRD

```
                                                    MetAlaLe
                           ggatccgatcttggaggatgattaaatggcctt
                           ggatccgatcttggaggatgattaaatggcctt

uAspCysThrArgValThrCysProGluGlyTrpLeuProPheGlnGlyLysCysTyrTy
agactgtaccagggtcacctgtcctgaaggctggctccctttcagggtaagtgttacta
agactgtaccagggtcacctgt

rPheSerProSerThrLysSerTrpAspGluAlaArgLysPheCysGlnGluAsnTyrSe
cttctctccaagcaccaagtcatgggatgaagcccggaagttctgccaggagaattactc

rHisLeuValIleIleSerAsnSerAspGluGlnAspPheValAlaLysAlaHisGlySe
tcacttggtcatcatcagtaactctgatgaacaggactttgtagccaaggctcacggatc

rProArgValTyrTrpLeuGlyLeuAsnAspArgAsnValGluGlyAspTrpArgTrpLe
tccacgggtgtactggctggggctgaatgacagaaatgtcgaaggggactggaggtggct

uAspGlySerProValThrLeuSerPheTrpAspProGlnGluProAsnAsnLeuTyrAs
ggatgggtcacctgtcacactgagcttttgggacccacaggaacccaacaacctctataa

nAsnGluAsnCysAlaSerMetAsnLysGlyGlyThrTrpAsnAspLeuSerCysAspLy
taatgagaactgtgccagcatgaacaaaggtggcacctggaatgacctctcctgtgacaa

sThrThrTyrTrpIleCysGluArgLysCysSerCysAlaLeuAsnAspIlePheGluAl
aaccacgtattggatttgtgagcggaaatgttcctgtgccctgaatgacatcttcgaagc
                acactcgcctttacaaggacacgggacttactgtagaagcttcg

aGlnLysIleGluTrpHisGlu***
acagaaaatcgagtggcatgagtag
tgtcttttagctcaccgtactcatc
```

Figure S16. CD23 CRD

```
                                    MetAlaAsnGlySerValCysAsn
           ggatccgatcttggaggatgattaaatggccaacggctctgtgtgcaac
           ggatccgatcttggaggatgattaaatggccaacggctctgtgtgcaac

ThrCysProGluAlaTrpIleTyrPheGlnLysLysCysTyrTyrPheGlyGluGlyAla
acgtgccccgaggcatggatctatttccaaaagaagtgctactacttcggggagggcgcc
acgtgc

LysLysTrpIleGlnAlaArgTyrAlaCysGluAsnLeuHisGlyArgLeuValSerIle
aagaaatggatccaggcccggtacgcctgtgaaaatctgcacgggcggctggttagcatc

HisSerProGluGluGlnAspPheLeuThrLysArgAlaAsnTrpArgGlySerTrpIle
cacagcccagaggagcaggacttcctgaccaaacgtgccaactggaggggctcctggatt

GlyLeuArgAspLeuAspIleGluGlyGluPheIleTrpMetAspAsnGlnProLeuAsp
ggccttcgggacctggacattgaaggggagtttatctggatggacaaccagcccctggac

TyrSerAsnTrpGlnProGlyGluProAsnAspAlaGlyGlnGlyGluAsnCysValMet
tatagcaactggcagccaggggagcccaacgatgcaggccagggtgagaactgcgtgatg

MetLeuGlySerGlyLysTrpAsnAspAlaPheCysGlySerGluLeuHisGlyTrpVal
atgctgggctctgggaagtggaatgacgccttctgtggaagcgaacttcatggctgggtg
                                                         cac

CysAspArgLeuAlaThrCysGlyLeuAsnAspIlePheGluAlaGlnLysIleGluTrp
tgcgaccggctggccacgtgcggcctgaatgacatcttcgaagcacagaaaatcgagtgg
acgctggccgaccggtgcacgccggacttactgtagaagcttcgtgtcttttagctcacc

HisGlu***
catgagtaggaattc
gtactcatccttaag
```

Figure S17. LSECtin CRD

```
                               MetAlaSerSerCysLysGluCysProGl
      ggatccgatcttggaggatgattaaatggccagctcctgcaaggagtgccccga
      ggatccgatcttggaggatgattaaatggccagctcctgcaaggagtgccccga

uSerTrpLeuProPheGlnGlySerCysTyrPhePheSerThrLeuArgAlaThrTrpVa
gtcgtggctgccgttccagggttcctgttacttcttctccacgctgcgggccacgtgggt
g

lGluAlaGlnGlnHisCysGluArgSerGlyAlaHisLeuValIleValGlyGlyLeuGl
ggaggcacagcagcactgcgagcgctccggcgcgcacctggtgatagtcggaggcctgga

uGluGlnGlyPheLeuSerArgAsnThrArgGlyArgGlyTyrTrpLeuGlyLeuArgAl
agagcagggtttcctgagtcggaatacgcgtggccgcggttattggctgggcctcagggc

aValArgLysValArgArgIleGlnGlyTyrGlnTrpValAspGlyValAlaLeuSerPh
cgtgcgcaaggtgcgcaggatccagggctaccagtgggtggacggagtcgcgctcagttt

eSerHisTrpAsnArgGlyGluProAsnAspSerMetGlyArgGluAspCysIleMetMe
cagccactggaatcgggggagcccaacgactctatggggcgcgaggattgtatcatgat

tLeuArgThrGlyMetTrpAsnAspAlaProCysAspAsnGluAsnAspAsnTrpIleCy
gctccgcacggggatgtggaacgacgcgccatgtgacaacgagaacgacaactggatctg
                                                   gacctagac

sGluLysArgLeuSerCysGlyLeuAsnAspIlePheGluAlaGlnLysIleGluTrpHi
tgagaagaggctcagctgcggtctgaatgacatcttcgaagcacagaaaatcgagtggca
actcttctccgagtcgacgccagacttactgtagaagcttcgtgtcttttagctcaccgt

sGlu***
tgagtagtt
actcatcaa
```

Figure S18. Endo180 CRD 2

```
                                                      MetAla
                             ggatccgatcttggaggatgattaaatggcc

ValGluCysGluProSerTrpGlnProPheGlnGlyHisCysTyrArgLeuGlnAlaGlu
gtggagtgtgagcccagctggcagcccttccagggccactgctaccgcctgcaggctgag

LysArgSerTrpGlnGluSerLysLysMetCysLeuArgGlyGlyGlyAspLeuLeuSer
aagcgcagctggcaggagtccaagaagatgtgtctgcggggtgggggcgacttgctcagc

IleHisSerMetAlaGluLeuGluPheIleThrLysGlnIleLysGlnGluValGluGlu
atccacagcatggcggagctggagttcatcaccaagcagatcaagcaggaggtggaggag

LeuTrpIleGlyLeuAsnAspLeuLysLeuGlnMetAsnPheGluTrpSerAspGlySer
ctgtggattggtctcaacgacctgaaactgcagatgaattttgagtggtccgacgggagc

LeuValSerPheThrHisTrpHisProPheGluProAsnAsnPheArgAspSerLeuGlu
ctcgtgagcttcacccattggcacccctttgagcccaacaacttccgagacagcctggag

AspCysValThrIleTrpGlyProGluGlyArgTrpAsnAspSerProCysAsnGlnSer
gactgtgtcaccatctgggggccggaaggtcgctggaacgacagtccctgtaaccagtcc

LeuProSerIleCysLysLysLeuAsnAspIlePheGluAlaGlnLysIleGluTrpHis
ctgccgtccatttgcaagaagctgaatgacatcttcgaagcacagaaaatcgagtggcat

Glu***
gagtaggaattc
```

Figure S19. Mincle CRD

```
                                                   aaggat
                                                   aaggat

                    MetAlaCysProLeuLysTrpPheHisPheGlnSerSer
ccgatcttggaggatgattaaatggcttgtccactgaagtggttccatttcaatccagc
ccgatcttggaggatgattaaatggcttgtccactgaagtggttccatttc

CysTyrLeuPheSerProAspThrMetSerTrpArgAlaSerLeuLysAsnCysSerSer
tgctacttatttctcctgacaccatgtcatggagagcaagtctaaaaaactgctcgagc

MetGlyAlaHisLeuValValIleAsnThrGlnGluGluGlnGluPheLeuTyrTyrThr
atgggtgctcacctggtggttatcaacacgcaggaggagcaggaattcctttactacaca

LysProArgLysLysGluPheTyrIleGlyLeuThrAspGlnValThrGluGlyGlnTrp
aaacctagaaagaaggagttttatattggactgacggaccaggtgactgagggtcagtgg

GlnTrpValAspGlyThrProPheThrLysSerLeuSerPheTrpAspAlaGluGluPro
caatgggtagatggtacacctttcacaaagtctctgagcttctgggatgcaggggagccc

AsnAsnLeuValThrValGluAspCysAlaThrIleArgAspSerSerAsnProArgGln
aacaacctggttactgtggaggactgtgccaccataagggactcctcaaatccaaggcaa

AsnTrpAsnAspValProCysPhePheAsnMetPheArgValCysGluMetProGluArg
aactggaatgatgtgccctgtttcttcaatatgtttcgggtttgtgaaatgccagaaaga
                                  gcccaaacactttacggtctttct

LysIleGlyLeuAsnAspIlePheGluAlaGlnLysIleGluTrpHisGlu***
aagattggactgaatgacatcttcgaagcacagaaaatcgagtggcatgagtagaagctt
ttctaacctgacttactgtagaagcttcgtgtcttttagctcaccgtactcatcttcgaa
```

Figure S20. Dectin-2 CRD

```
                                                        ggat
                                                        ggat

                    MetAlaLeuThrCysPheSerGluGlyThrArgValThr
ccgatcttggaggatgattaaatggccctaacctgcttcagtgaagggacaagggtgaca
ccgatcttggaggatgattaaatggccctaacctgcttcagtgaagggaca

GluLysIleTrpGlyCysCysProGlyThrTrpLysProPheGlySerSerCysTyrPhe
gaaaagatttggggatgttgcccaggtacctggaagccgtttggttccagctgctacttt

IleSerSerGluGluAsnPheTrpAlaLysSerGluGlnAsnCysIleGlyMetGlyAla
atttcttctgaagagaatttctgggctaagagtgagcagaactgcattgggatgggagct

HisLeuValValIleAsnThrGluThrGluGlnAspPheIleIleGlnGlnLeuAsnLys
cacttggtggtgatcaacacagaaacagagcaggatttcattatccagcagctgaataaa

ThrPheSerTyrPheLeuGlyLeuSerAspProGlnGlyAsnGlyAsnTrpGlnTrpIle
acatttcttattttctgggactctcagacccacaagggaatggcaactggcaatggatt

AspGlnThrProTyrLysGluAsnValArgPheTrpHisGlnAsnGluProAsnPheSer
gatcagacaccttacaaggaaaatgtcagattttggcaccaaaatgaacccaactttct

AlaGluGluCysAlaSerValValPheTrpAspGlyArgGlyTrpGlyTrpAsnAspVal
gcagaggaatgtgcttcagttgttttctgggatgggagaggatggggctggaatgatgtt

PheCysAspSerLysArgLysSerIleCysGluMetLysLysIleTyrLeuLeuAsnAsp
ttctgtgattctaaaaggaaatcaatatgtgagatgaagaagatttacctactgaatgac
                          acactctacttcttctaaatggatgacttactg

IlePheGluAlaGlnLysIleGluTrpHisGlu***
atcttcgaagcacagaaaatcgagtggcatgagtaggaattc
tagaagcttcgtgtcttttagctcaccgtactcatccttaag
```

Figure S21. Dectin-1 wild type extracellular region (Dectin-1 WT)

```
                                     MetAlaLeuAsnAspIlePheGl
                                  taaatggccctgaatgacatcttcga

uAlaGlnLysIleGluTrpHisGluGlySerGlyIleTrpArgSerSerSerGlyAsnAs
agcacagaaaatcgagtggcatgaaagatctggtatttggagatccagttcagggaacaa
                       aaagatctggtatttggagatccagttcagggaac

nLeuLeuLysSerAspSerPheProSerArgAsnLysAspAsnGlnSerGlnProThrGl
tctgttgaagagtgacagctttccatcaagaaataaagacaaccagagtcaacccacaca

nSerSerLeuGluAspSerValIleProThrLysAlaLeuThrThrThrGlyValPheSe
atcatctttagaagatagtgtgatacctaccaaggctctcacgaccacaggagttttctc

rSerSerCysProProAsnTrpIleThrHisGluAspSerCysTyrLeuPheSerThrLe
tagctcttgtcccccctaactggatcacacatgaggatagctgttatctatttagcacact

uLeuAspSerTrpAspGlySerLysArgGlnCysPheGlnLeuGlySerHisLeuLeuLy
attagattcctgggatggaagtaaaagacaatgctttcaactgggctctcatctcctgaa

sIleAspSerSerLysGluLeuGluPheIleSerArgGlnValSerSerGlnProAspHi
gatagacagctcaaaagagttggagtttatatcaaggcaagtgtcttcccagcctgatca

sSerPheTrpIleGlyLeuSerArgArgGlnThrGluGluProTrpLeuTrpGluAspGl
ttcattttggatagggctttctcgccgtcagacagaagaaccatggctctgggaggatgg

ySerThrLeuLeuSerAsnLeuPheGlnIleArgSerThrValThrGluLysAspSerSe
ctccaccttgttgtctaacctgttccaaatcagaagtacagttaccgaaaaagactcatc

rHisAsnCysAlaTrpIleHisValSerAspIleTyrAspGlnLeuCysSerValHisSe
tcacaactgtgcatggatccatgtgtcagacatttacgaccaactttgtagtgtgcattc

rTyrSerIleCysGluLysLysLeuSerVal***
atacagtatttgtgagaagaagttgtcagtataaaagggc
                ttcttcaacagtcatattttcccg
```

Figure S22. Dectin-1 mutant extracellular region (Dectin-1 Mut)

```
                                MetAlaLeuAsnAspIlePheGl
                             taaatggccctgaatgacatcttcga

uAlaGlnLysIleGluTrpHisGluGlySerGlyIleTrpArgSerSerSerGlyAnAs
agcacagaaaatcgagtggcatgaaagatctggtatttggagatccagttcagggaacaa
                       aaagatctggtatttggagatccagttcagggaac

nLeuLeuLysSerAspSerPheProSerArgAsnLysAspAsnGlnSerGlnProThrGl
tctgttgaagagtgacagctttccatcaagaaataaagacaaccagagtcaacccacaca

nSerSerLeuGluAspSerValIleProThrLysAlaLeuThrThrThrGlyValPheSe
atcatctttagaagatagtgtgatacctaccaaggctctcacgaccacaggagttttctc

rSerSerCysProProAsnTrpIleThrHisGluAspSerCysTyrLeuPheSerThrLe
tagctcttgtccccctaactggatcacacatgaggatagctgttatctatttagcacact

uLeuAspSerTrpAspGlySerLysArgGlnCysPheGlnLeuGlySerHisLeuLeuLy
attagattcctgggatggaagtaaaagacaatgctttcaactgggctctcatctcctgaa

sIleAspSerSerLysGluLeuGluPheIleSerArgGlnValSerSerGlnProAspHi
gatagacagctcaaaagagttggagtttatatcaaggcaagtgtcttcccagcctgatca

sSerPheTrpIleGlyLeuSerArgArgGlnThrGluGluProTrpLeuTrpGluAspSe
ttcattttggatagggctttctcgccgtcagacagaagaaccatggctctgggaggatag

rSerThrLeuLeuSerAsnLeuPheGlnIleArgSerThrValThrGluLysAspSerSe
ctccaccttgttgtctaacctgttccaaatcagaagtacagttaccgaaaaagactcatc

rHisAsnCysAlaTrpIleHisValSerAspIleTyrAspGlnLeuCysSerValHisSe
tcacaactgtgcatggatccatgtgtcagacatttacgaccaactttgtagtgtgcattc

rTyrSerIleCysGluLysLysLeuSerVal***
atacagtatttgtgagaagaagttgtcagtataaaagggc
                ttcttcaacagtcatattttcccg
```

Figure S23. Asialoglycoprotein Receptor subunit 1 CRD (ASGPR1)

```
                                          MetAlaSerCysGlnMet
                 ggatccgatcttggaggatgattaaatggccagctgtcagatg
                 ggatccgatcttggaggatgattaaatggccagctgtcagatg

AlaValLeuGlnGlyAsnGlySerGluLysAlaCysCysProValAsnTrpIleAspTyr
gctgtcctccagggcaatggctctgaaaaagcctgctgcccagtgaactggatcgattat
gctgtcctccag

GluGlySerCysTyrTrpPheSerArgSerGlyLysProTrpProGluAlaGluLysTyr
gaaggcagctgttactggttctctcgctccgggaagccctggccagaggctgagaagtac

CysGlnLeuGluAsnAlaHisLeuValValValGlySerTrpGluGluGlnLysPheIle
tgccagttggagaatgcccacctggtggtggtgggctcctgggaggagcagaaatttatc

GlnHisHisMetGlyProValAsnThrTrpIleGlyLeuMetAspGlnAsnGlyProTrp
cagcaccacatgggccctgtaaatacctggataggcctcatggatcaaaatgggccctgg

LysTrpValAspGlyThrAspTyrGluThrGlyPheLysAsnTrpAlaProGluGlnPro
aaatgggtggacgggacggactacgagacgggcttcaagaactgggcaccagagcagcca

AspAspTrpTyrGlyHisGlyLeuGlyGlyGlyGluAspCysAlaHisIleThrValAsp
gatgactggtatgggcatgggctcggagggggtgaagactgtgcccacatcacggtggac

GlyArgTrpAsnAspAspValCysLeuArgProTyrArgTrpValCysGluAlaGlnArg
ggccgctggaatgatgacgtctgcctgaggccctaccgctgggtctgtgaggcccagcgg
                                                      gtcgcc

AspGlyGlyAsnAspSerLeuAsnAspIlePheGluAlaGlnLysIleGluTrpHisGlu
gacggaggcaatgacagcctgaatgacatcttcgaagcacagaaaatcgagtggcatgag
ctgcctccgttactgtcggacttactgtagaagcttcgtgtcttttagctcaccgtactc

***
taggaattc
atccttaag
```

Figure S24. Asialoglycoprotein Receptor subunit 2 CRD (ASGPR2)

```
                                           MetAlaThrCysGlnMetAla
                                           atggccacttgtcagatggca
                                            tggccacttgtcagatggca

HisPheGlnSerAsnGlyThrGluCysCysProValAsnTrpValAspHisAspGlySer
cacttccagagcaatggcacagaatgctgcccagtgaactgggtggaccatgatggcagc
cacttccag

CysTyrTrpPheSerArgSerGlyLysProTrpLeuGluAlaGluLysTyrCysGlnLeu
tgctactggttctctcgctcagggaagccctggctcgaggctgagaagtactgccagctg

GluAsnAlaHisLeuValValIleAsnSerArgGluGluGlnLysPheIleValGlnHis
gagaatgcccacctcgtggtcatcaactccagagaggaacagaagttcattgtacaacac

ThrAsnProPheArgValTrpIleGlyLeuThrAspSerAspGlySerTrpLysTrpVal
acaaacccctttagagtctggataggtctcactgacagcgatggctcctggaaatgggtg

AspGlyThrAspTyrLysHisSerTyrLysAsnTrpAspProAlaGlnProAspAspTrp
gacggcacagactacaagcacagctacaagaactgggatcccgctcagcccgatgactgg

ArgGlyHisGluLeuGlyAlaSerGluAspCysAlaGluIleArgTrpAspGlyArgTrp
cgggggcacgagctgggggccagcgaggactgtgcagagatcagatgggatgggcgctgg

AsnAspAspPheCysGlnGlnValLysArgTrpValCysGluThrLysArgAsnIleThr
aatgacgatttctgccagcaagtgaaacgctgggtgtgtgagacaaagcggaacatcacc
                                           ctctgtttcgccttgtagtgg

MetLeuAsnAspIlePheGluAlaGlnLysIleGluTrpHisGlu***
atgctgaatgacatcttcgaagcacagaaaatcgagtggcatgagtaggttaag
tacgacttactgtagaagcttcgtgtcttttagctcaccgtactcatccttaag
```

Figure S25. Macrophage Galactose Receptor CRD (MGL)

```
                                                         Met
                                ggatccgatcttggaggatgattaaatg
                                ggatccgatcttggaggatgattaaatg

AlaLeuThrCysLysMetAspAlaLeuLysSerAsnGlySerGlnAsnThrAlaCysCys
gccctgacctgcaagatggatgctctcaagagcaatggctctcaaaacacagcctgctgt
gccctgacctgcaagatggatgctctc

ProAlaAsnTrpLeuGluHisGluGlyHisCysTyrTrpPheSerSerLeuArgLysPro
cccgccaactggctggagcatgaaggccactgctactggttctcctccttgcggaagccc

TrpProGluAlaGluLysAspCysGlnLeuLysAsnAlaGlnLeuValValIleAsnSer
tggccagaagctgagaaagactgccaactgaagaatgcccaactggtagtcatcaactcc

ArgAspGluGlnAspPheIleGlnAlaAsnLeuHisProTyrPheThrTrpMetGlyLeu
agagacgaacaggattttatccaggccaacctacatccttacttcacctggatgggcctc

SerAspProAspGlyValTrpLysTrpValAspGlySerAspTyrGluThrAsnIleLys
agtgatccggatggagtctggaaatgggtggatgggtcggactatgagaccaacatcaag

AsnTrpLysProGlyGlnProAspAspPheHisGlyHisGlyLeuGlyGlyGlyGluAsp
aattggaagccaggccagccggatgactttcatgggcatgggctgggtgggggtgaggac

CysAlaHisPheTyrProAspGlyGluTrpAsnAspAspAlaCysGlnArgLeuTyrTyr
tgtgcccatttctaccctgatggcgagtggaatgacgatgcctgccaaagactctactac

TrpIleCysGluAlaGlyLeuSerGlnValHisAsnLysMetHisLeuAsnAspIlePhe
tggatctgcgaggctggactgagccaagtacacaataaaatgcacctgaatgacatcttc
               cctgactcggttcatgtgttattttacgtggacttactgtagaag

GluAlaGlnLysIleGluTrpHis***
gaagcacagaaaatcgagtggcatgagtaggaattc
cttcgtgtcttttagctcaccgtactcatccttaag
```

Figure S26. Scavenger Receptor C-type Lectin CRD (SRCL)

```
                                   ggatccgatcttggaggatgattaa
                                   ggatccgatcttggaggatgattaa

MetAlaGluAspAsnGlyCysLeuProTyrTrpLysAsnPheThrAspLysCysTyrTyr
atggccgaggacaacggctgcctgccttactggaagaacttcacagacaaatgctactat
atggccgaggacaacggctgcctgccttac

PheSerThrGluArgAspPhePheGluAspAlaLysLeuPheCysGluArgMetSerSer
ttttcaactgagagagactttttgaggatgcaaaactttctgcgaaagaatgtcttca

HisLeuValPheIleAsnThrGlyGluGluGlnGlnTrpIleLysAsnGlnMetValAla
catctcgttttcataaacacgggagaggagcagcaatggatcaaaaaccagatggtggcg

LysGlnAsnTyrTrpIleGlyLeuThrAspLeuGluGlnGluAsnGluTrpArgTrpLeu
aaacagaactactggattggcctcacggacttggagcaggagaatgaatggaggtggctg

AspGlyThrLeuLeuGluTyrLysAsnTrpLysAlaGlyGlnProAspAsnTrpGlyHis
gatgggacacttctggagtacaaaaattggaaagctggacagccagataactggggtcat

GlyHisGlyProGlyGluAspCysAlaGlyLeuIleAsnPheGlyGlnTrpAsnAspPhe
ggccacgggcctggagaagattgcgctggactgattaattttgggcagtggaacgatttc

ProCysGluAspMetAsnHisPheIleCysGluLysAspArgGluArgGluLeuAlaIle
ccatgtgaagacatgaatcacttcatctgcgagaaagacagggagagagaattagcaatt
                                      ctgtccctctcttaatcgttaa

ThrLeuLeuAsnAspIlePheGluAlaGlnLysIleGluTrpHisGlu***
acattactgaatgacatcttcgaagcacagaaaatcgagtggcatgagtaggaattc
tgtaatgacttactgtagaagcttcgtgtcttttagctcaccgtactcatccttaag
```

Figure S27. Kupffer Cell Receptor CRD (KCR CRD)

```
                                                      MetAla
                             ggatccgatcttggaggatgattaaatggcc
                             ggatccgatcttggaggatgattaaatggcc

TyrSerGlySerLeuTyrTyrPheSerSerAlaLysLysThrTrpGlnGluAlaGluGln
tacagtgggagcttgtattacttttcttctgccaagaagacgtggcaggaggccgagcag
tacagtgggagcttgtattacttttc

PheCysValSerHisGlyAlaHisLeuAlaSerValThrSerGluGluGluLysThrPhe
ttctgtgtgtcccatggagcccacctggcctcggtgacctcggaggaggagaagacattt

LeuIleGlnPheThrSerSerValTyrHisTrpIleGlyLeuThrAspHisGlyThrGlu
ctgatacagttcacgagttctgtttaccactggattggcctcactgaccacggtacggag

GlyHisTrpArgTrpThrAspGlyThrAlaPheAspArgAlaArgSerArgAlaPheTrp
ggccactggcgctggacagatggcacagcattcgatcgtgccaggagccgtgcgttttgg

AlaGluAsnGlnProAspAsnTrpGlnHisGlyIleGlyGlnSerGluAspCysValGln
gctgagaatcagccagataactggcaacacggtattgggcaatcggaagactgtgtccag

MetGlnGlnLysTrpAsnAspIleSerCysSerThrLeuCysArgTrpIleCysLysLys
atgcagcagaagtggaatgacatatcctgctccactctctgccgctggatctgcaagaag
                                                   acgttcttc

ProMetValGlnLeuLeuAsnAspIlePheGluAlaGlnLysIleGluTrpHisGlu***
cctatggtccagctgctgaatgacatcttcgaagcacagaaaatcgagtggcatgagtag
ggataccaggtcgacgacttactgtagaagcttcgtgtcttttagctcaccgtactcatc
```

Figure S28. Kupffer Cell Receptor extracellular region (KCR ECD)

```
                                        MetAlaLeuAsnAspIlePh
                                        atggccctgaatgacatctt

eGluAlaGlnLysIleGluTrpHisGluGlySerThrGlnThrPheValArgGlySerLe
cgaagcacagaaaatcgagtggcatgagggatccacccagacctttgtaagaggcagttt
                            ggatccacccagacctttgtaagaggcagt

uAspAsnThrSerAlaGlnIleGlnValLeuArgSerHisLeuGluArgAlaGlyGlyGl
agacaacaccagtgctcagatccaggtgttaagaagtcatttggaaagggctggaggtga

uIleHisLeuLeuLysArgAspLeuGluAsnValThrAlaGlnThrGlnThrAlaSerSe
gattcacttgttaaaaagagatttggaaaatgtcactgcccagacccaaacagcaagcag

rHisLeuGluGlnThrAspAlaGluMetArgValLeuLysThrGluLeuGluSerAlaIl
tcacctggagcagacagatgctgagatgagagtattaaaaacagagctggaaagtgccat

eAlaLeuSerSerLysIleGlnValLeuAsnGlyLeuLeuArgAsnAlaSerGlnGluIl
tgccttaagttccaagattcaggtgttaaatggtcttttgagaaatgccagccaagagat

eGlnThrLeuLysGlnGlyMetLysAspAlaAlaAlaLeuGlnSerGlnThrGlnMetLe
acagaccttaaaacaaggaatgaaggatgccgcagccttacagtcccagacccaaatgtt

uGluArgSerLeuGlnGluAlaArgThrGluIleGlnThrLeuArgLysAspLeuGlyAs
agagagaagtctgcaggaggccagaactgagatccagacattaagaaaggatttggggaa

nThrLysThrLeuArgThrThrIleGlnGluGlnGlnArgSerLeuGluSerPheArgTh
caccaaaacactgaggacaacaatccaggagcagcagagaagcctggagtccttccgcac

rAlaLeuAlaSerGlnGluGlnLeuGlnArgAsnHisAsnGlnLeuPheGlnLeuPheLe
agccttggcttcacaggagcagctccagaggaaccacaatcaacttttccagctgttcct

uGlnGlyTrpLysPheTyrSerGlySerLeuTyrTyrPheSerSerAlaLysLysThrTr
gcaaggctggaagttctacagtgggagcttgtattactttcttctgccaagaagacgtg

pGlnGluAlaGluGlnPheCysValSerHisGlyAlaHisLeuAlaSerValThrSerGl
gcaggaggccgagcagttctgtgtgtcccatggagcccacctggcctcggtgacctcgga

uGluGluLysThrPheLeuIleGlnPheThrSerSerValTyrHisTrpIleGlyLeuTh
ggaggagaagacatttctgatacagttcacgagttctgtttaccactggattggcctcac

rAspHisGlyThrGluGlyHisTrpArgTrpThrAspGlyThrAlaPheAspArgAlaAr
tgaccacggtacggagggccactggcgctggacagatggcacagcattcgatcgtgccag

gSerArgAlaPheTrpAlaGluAsnGlnProAspAsnTrpGlnHisGlyIleGlyGlnSe
gagccgtgcgttttgggctgagaatcagccagataactggcaacacggtattgggcaatc

rGluAspCysValGlnMetGlnGlnLysTrpAsnAspIleSerCysSerThrLeuCysAr
ggaagactgtgtccagatgcagcagaagtggaatgacatatcctgctccactctctgccg

gTrpIleCysLysLysProMetValGlnVal***
ctggatctgcaagaagcctatggtccagctgtag
        cgttcttcggataccaggtcgacatc
```

Table S1
Bacterial strains used in this study.

Bacterial strain	Serotype	Plasmid
Enteropathogenic *E. coli* strain E2348/69	O127:H6	pACYC184-GFP
Enterohaemorrhagic *E. coli* strain EDL933	O157:H7	pUltra-GFP/Gm
K. pneumoniae strain 43816	K2:01	pUltra-GFP/Gm
K. pneumoniae strain B5055	K2:O1	pUltra-GFP/Gm
K. pneumoniae strain B5055nm	K-:O1	pUltra-GFP/Gm
E. coli strain BL21(DE3)		pET28a-eGFP
E. coli strain ClearColi		pET28a-eGFP
E. coli strain K12		-
S. aureus strain Wood 46		-
M. bovis strain bacillus Calmette-Guerin Pasteur Δ*panCD*		pCB22-Turbo635-ASV-YFP
